# Determinants beyond Both Complementarity and Cleavage Govern MicroR159 Efficacy in *Arabidopsis*

**DOI:** 10.1371/journal.pgen.1004232

**Published:** 2014-03-13

**Authors:** Junyan Li, Marlene Reichel, Anthony A. Millar

**Affiliations:** Research School of Biology, Australian National University, Acton, Australian Capital Territory, Australia; University of California Riverside, United States of America

## Abstract

Plant microRNAs (miRNAs) are critical regulators of gene expression, however little attention has been given to the principles governing miRNA silencing efficacy. Here, we utilize the highly conserved *Arabidopsis* miR159-*MYB33/MYB65* regulatory module to explore these principles. Firstly, we show that perfect central complementarity is not required for strong silencing. Artificial miR159 variants with two cleavage site mismatches can potently silence *MYB33/MYB65*, fully complementing a loss-of-function *mir159* mutant. Moreover, these miR159 variants can cleave *MYB33*/*MYB65* mRNA, however cleavage appears attenuated, as the ratio of cleavage products to full length transcripts decreases with increasing central mismatches. Nevertheless, high levels of un-cleaved *MYB33*/*MYB65* transcripts are strongly silenced by a non-cleavage mechanism. Contrary to *MIR159a* variants that strongly silenced endogenous *MYB33*/*MYB65*, artificial *MYB33* variants with central mismatches to miR159 are not efficiently silenced. We demonstrate that differences in the miRNA:target mRNA stoichiometry underlie this paradox. Increasing miR159 abundance in the *MYB33* variants results in a strong silencing outcome, whereas increasing *MYB33* transcript levels in the *MIR159a* variants results in a poor silencing outcome. Finally, we identify highly conserved nucleotides that flank the miR159 binding site in *MYB33*, and demonstrate that they are critical for efficient silencing, as mutation of these flanking nucleotides attenuates silencing at a level similar to that of central mismatches. This implies that the context in which the miRNA binding site resides is a key determinant in controlling the degree of silencing and that a miRNA “target site” encompasses sequences that extend beyond the miRNA binding site. In conclusion, our findings dismiss the notion that miRNA:target complementarity, underpinned by central matches, is the sole dictator of the silencing outcome.

## Author Summary

In plants, microRNAs (miRNAs) are critical regulators of gene expression. As most validated targets are of high complementarity, whose transcripts are cleaved by the miRNA, both complementarity and cleavage are thought to be the major factors determining the degree to which a target gene is silenced. Here, we explore this principle utilizing the highly conserved miR159-*MYB33/MYB65* regulatory module in the model flowering plant *Arabidopsis*. Firstly, we demonstrate that perfect central complementarity facilitates efficient transcript cleavage but is not required for a strong silencing outcome, as miR159 variants with two central mismatches can recognize and silence *MYB33/MYB65* effectively *in planta*. Driving this silencing is a potent miR159-mediated non-cleavage mechanism that ensures total silencing even when *MYB33* transcript levels are very high. Secondly, we demonstrate that the stoichiometric ratio of miRNA to target mRNA is a critical determinant of a silencing outcome, and that ratio becomes increasingly important for inefficient miRNA-target interactions. Finally, we show that nucleotides flanking the miR159 binding site of *MYB33* are essential for efficient silencing, demonstrating that the sequence context in which the miRNA target site resides in has a major impact on the silencing outcome. Together, we have shown that although high complementarity underpinned by efficient transcript cleavage may be a prerequisite for a strong silencing outcome, many additional factors that modulate the strength of the miRNA-target interaction are at play. These findings will have ramifications for bioinformatics prediction of miRNA targets and design of artificial miRNAs.

## Introduction

Ubiquitously found in plants and animals, microRNAs (miRNAs) are a group of 20–24 nucleotide (nt) small RNAs (sRNAs) that have been demonstrated to be critical regulators of gene expression. In plants, there are hundreds of known miRNAs [1], many of which have been shown to play critical roles in many different developmental and physiological processes [2]. They silence gene expression by guiding the RNA induced silencing complex (RISC) to target mRNAs via base pairing [2]. In plants, the subsequent repression of the target transcript occurs mainly through mRNA cleavage and/or translational inhibition [3].

Plant miRNAs recognise highly complementary binding sites, which enabled accurate bioinformatics prediction of their targets [4]. Moreover, experimentally determined targets were defined by the landmark study of Schwab et al. (2005), formulating the empirical parameters governing miRNA target recognition in plants, which are based purely on complementarity between the miRNA-target mRNA pairs [5]. It was found that there should be no more than one mismatch in the important “seed region” which corresponds to the 5′ end of the miRNA from positions 2 to 12, including no mismatches at the cleavage site (positions 10 and 11), and no more than two contiguous mismatches in the 3′ of the miRNA (positions 13–21) [5], [6]. Although there are examples of miRNA targets that do not fully conform to these sequence parameters [4], [7], generally most experimentally validated miRNA targets satisfy these parameters, and have been verified through degradome sequencing [8], [9]. Consequently, these parameters have been widely accepted and incorporated into many bioinformatic programs that predict miRNA targets [10].

Despite this extensive analysis on the complementary requirements for miRNA target recognition, and the subsequent mechanism of miRNA mediated repression [11], there has been very little analysis on the factors that impact the strength or efficacy of miRNA-mediated gene silencing in plants. It has been clearly shown that alterations in complementarity between the miRNA and its target can impact on the strength of the silencing outcome [12]. Additionally, the abundance of the miRNA can also functionally impact the silencing outcome [13], but generally no other factors have been considered in plants. As most plant miRNA-mRNA duplexes have high complementarity with perfect central pairing, transcript cleavage is generally considered a key silencing mechanism which results in the irreversible destruction of the transcript. This has been hypothesised to clear target transcript, leading to an absolute silencing outcome [14]. However, some plant miRNA targets with near perfect complementarity appear regulated primarily at the translational level [15]–[17], and this mechanism is likely widespread in plants [18]. Unlike cleavage, translational repression is thought not to result in mRNA destruction and thus is potentially reversible [3]. In animals, rather than an absolute silencing or “switch” mechanism, it acts as a tuning mechanism [19], however whether this is the case in plants is unclear. Due to this combinatorial mechanistic complexity, it is generally very difficult to appraise the strength of any miRNA-target interaction. Contributing to this in plants has been the lack of easily scorable systems that can gauge the extent of silencing. Consequently, the efficacy of plant miRNA-mediated regulation has remained largely ignored.

In this paper, we have used the highly conserved *Arabidopsis* miR159-*GAMYB* regulatory module to address fundamental questions regarding plant miRNA-mediated gene silencing. The *Arabidopsis* miR159 family is composed of three genes, *MIR159a*, *MIR159b* and *MIR159c*, although the *MIR159c* gene appears to be obsolete [20], [21]. Both miR159a and miR159b are predicted to regulate over 20 target genes in *Arabidopsis*, however regulation of only two targets appears physiologically important; namely *MYB33* and *MYB65*, two redundant genes encoding R2R3 MYB transcription factors [20], [21]. MiR159a and miR159b redundantly silences these two genes in rosettes, and perturbation of their activity enables *MYB33/MYB65* expression and results in a decreased rosette size and upwardly curled leaves [20], [22]. This easily scorable phenotype, along with molecular indicators, enables accurate appraisal of miR159 silencing efficiency.

Here, we complement a strong loss-of-function *mir159ab* double mutant with different artificial miR159 variants to demonstrate that perfect central complementarity is not required for the potent silencing of *MYB33* and *MYB65*. Instead, a strong non-cleavage repression mechanism was evident in transgenic plants accumulating high levels of *MYB33* transcripts, and appears to be a major contributing factor behind the strong efficacy of miR159. Finally, we show that factors beyond complementarity are critical for the silencing outcome. Firstly, the stoichiometric ratio of the miRNA:target mRNA strongly impacts the silencing outcome, especially when target cleavage is compromised. Moreover, mutation of sequences flanking the miR159 binding site of *MYB33* leads to an attenuation of silencing that is comparable to cleavage site mutations. This reveals an important role for binding site context in plant miRNA-mediated gene regulation, suggesting, in essence, that a miRNA “target site” encompasses sequences that extend beyond the complementary motif to which a miRNA directly binds.

## Results

### Artificial miR159a variants with up to two central mismatches can fully complement the *mir159ab* mutant

The *mir159ab* double mutant presents itself as an ideal tool to examine miRNA-mediated gene silencing, as all *mir159ab* defects are specifically caused by the deregulation of the redundant *GAMYB-like* gene pair *MYB33/MYB65*, and these mutant phenotypes, including leaf curliness and dwarfed stature, are easily scored [20]. Therefore, by complementing *mir159ab* with different miR159 variants with modified central complementarity to *MYB33/MYB65*, the impact of central pairing on the silencing outcome of miR159 or their variants can be evaluated.

**Figure 1:**
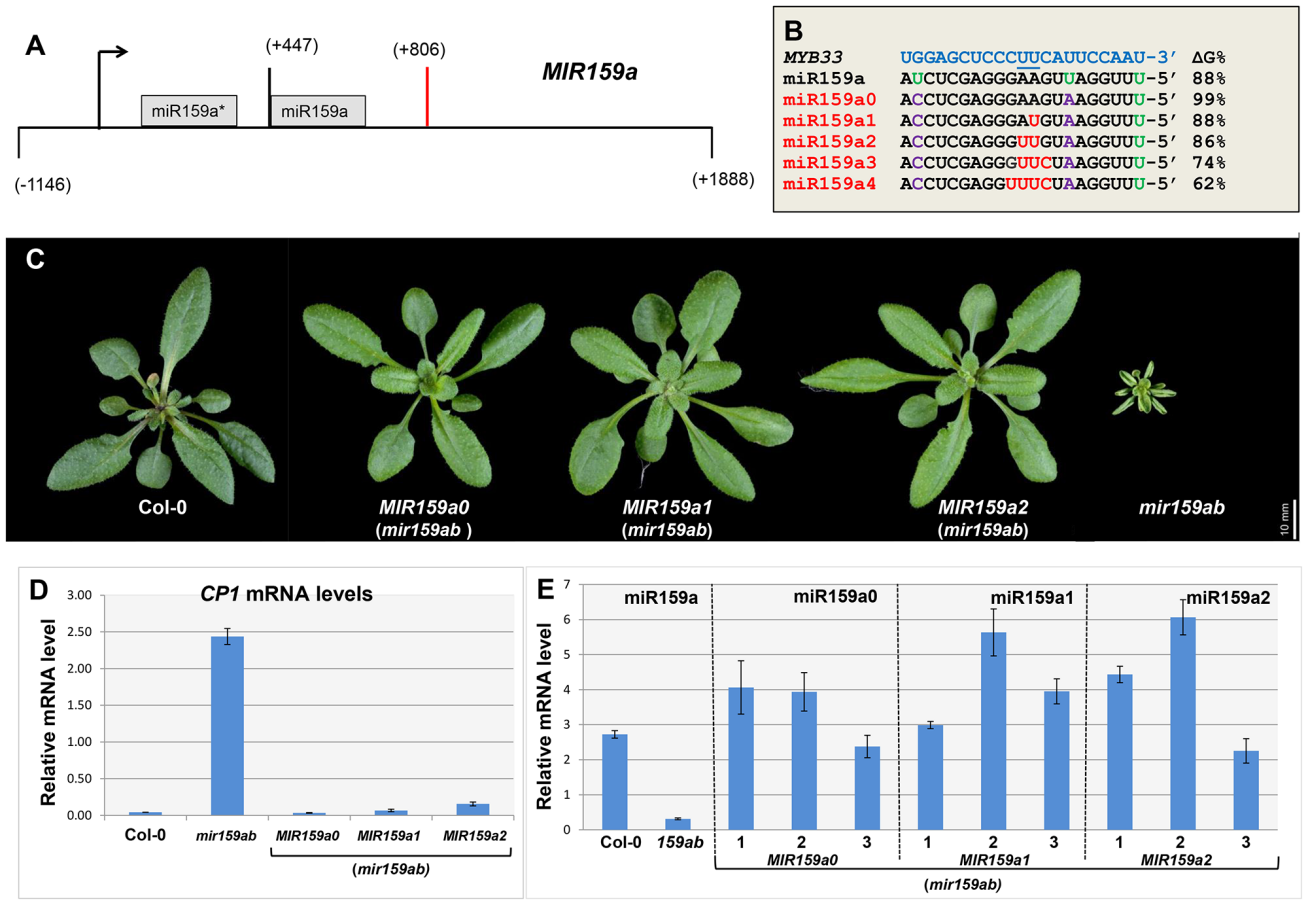
Artificial miR159a variants with up to two central mismatches are potent silencers of *MYB33/MYB65*. (A) Structure of the *MIR159a* transgene used to generate miR159a variant constructs. Numbers indicating relative positions to the transcription start site. Black arrow: Transcription start site; Orange bar: Polyadenylation site. (B) The alignment between *MYB33* and the miR159a variant sequences used to transform *mir159ab*. The percentage of free energy pairing with *MYB33* compared to a perfect match is listed on the right. Green: original mismatches; Purple: corrected mismatches; Red: introduced mismatches. (C) Aerial view of rosettes from 30-day-old *MIR159a0*, *MIR159a1* and *MIR159a2* plants (*mir159ab*) grown side by side with Col-0 and *mir159ab* under long day conditions. Scale bar = 10 mm. (D) The mRNA levels of *CP1* in the rosettes of *mir159ab* plants complemented by the *MIR159* variants in comparison with wild type and *mir159ab*. The value for each variant is the average of three independent transgenic lines. All mRNA levels were normalized with *CYCLOPHILIN 5*. Error bars represent the SEM. (E) Mature miR159a levels in Col-0 and *mir159ab*, and mature miR159a variant levels in three independent lines of *MIR159a0* (*mir159ab*), *MIR159a1* (*mir159ab*) and *MIR159a2* (*mir159ab*) transformants. All miR159a variant and miR159a levels were normalized with *sno101* by comparative quantitation analysis. Error bars represent the SEM.

Since miR159a has the highest complementarity to *MYB33/MYB65* and is the most abundant miR159 family member [23], five *MIR159a* variant constructs were generated by performing site-directed mutagenesis on a *MIR159a* genomic clone (Figure 1A). The two original mismatches of miR159a to *MYB33/MYB65* at positions 7 and 20 were corrected, to keep the miRNA-target pairing free energy of all variants as high as possible (Figure 1B). All five variants were identical except for their number of central mismatches to *MYB33*/*MYB65*, which ranged from zero to four. MiR159a0 was included as a positive control to ensure that changes at nucleotide positions 7 and 20 would not abolish target silencing, since these two mismatches are highly conserved between miR159 and the binding site of potential *GAMYB* target genes from different monocotyledonous and dicotyledonous species (Figure S1). For all constructs, compensatory mutations were made to the miR159a* sequence to maintain the original predicted *MIR159a* stem-loop secondary structure (Figure S2).

The five *MIR159a* variant constructs were individually transformed into *mir159ab* and the phenotypes of multiple primary transformants were scored. Correction of the mismatches at positions 7 and 20 had no influence on the silencing outcome, as all 30 *MIR159a0* T1 plants were fully complemented (Figure 1C). Surprisingly, all 44 *MIR159a1* and all 46 *MIR159a2* primary transformants obtained appeared fully wild type (Figure 1C), suggesting that mismatches at the cleavage site does not prevent silencing. In contrast, all 90 *MIR159a3* and 71 *MIR159a4* primary transformants appeared indistinguishable from *mir159ab* (data not shown), indicating that the introduction of more than two central mismatches between miR159 and *MYB33/MYB65* prevents complementation of the *mir159ab* phenotype.

To confirm that these *mir159ab* plants were fully complemented by the *MIR159a1* and *MIR159a2* variants, the expression of the *GAMYB-like* downstream gene *CYSTEINE PROTEINASE1* (*CP1*, AT4G36880) was examined by qRT-PCR, as its mRNA is highly expressed in *mir159ab* due to the deregulation of *MYB33/MYB65* [22]. Consistent with the morphological phenotypes, the mRNA level of *CP1* in *mir159ab* plants complemented by *MIR159a0*, *MIR159a1* or *MIR159a2* was close to that of wild-type, indicating that *MYB33/MYB65* expression was being suppressed to approximately wild type levels by these variants (Figure 1D). Next, we measured the abundance of the mature miR159a variants in complemented *mir159ab* plants using customized TaqMan sRNA assays. The levels of mature miR159a0, miR159a1 and miR159a2 in multiple independent transgenic lines were within the same order of magnitude as miR159a in wild type (Figure 1E). This suggests that gross overexpression of the miR159 variants was not an explanation for silencing targets with two central mismatches. Hence, their silencing potency in this regard would not be considered a transgenic artefact.

### MiR159a variants with up to two central mismatches can repress mRNA levels through a mechanism that includes transcript cleavage

**Figure 2:**
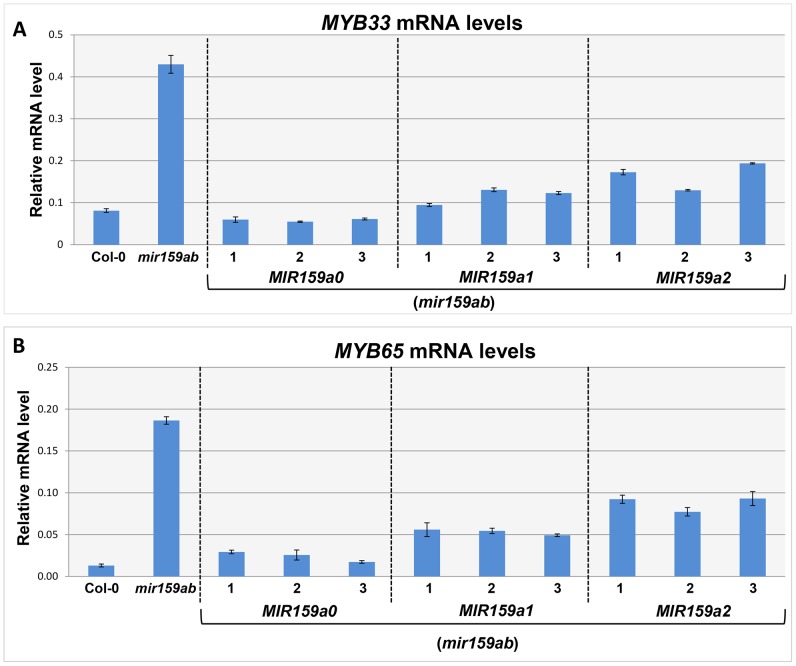
MiR159a1 and miR159a2 repress the endogenous *MYB33/MYB65* mRNA levels. (A) Un-cleaved *MYB33* mRNA levels in three independent lines of *MIR159a0* (*mir159ab*), *MIR159a1* (*mir159ab*) and *MIR159a2* (*mir159ab*) transformants; (B) Un-cleaved *MYB65* mRNA levels in three independent lines of *MIR159a0* (*mir159ab*), *MIR159a1* (*mir159ab*) and *MIR159a2* (*mir159ab*) transformants. All mRNA levels were normalized with *CYCLOPHILIN 5*. Measurements are the average of three technical replicates. Error bars represent the SEM. Similar results were obtained with two independent biological replicates.

In both animal and plants, it is a popular notion that miRNA-guided cleavage of target genes is significantly attenuated by central mismatches [24], [25]. Hence, to investigate the mechanism by which the miR159a1 and miR159a2 variants are silencing *MYB33* and *MYB65*, we firstly measured their un-cleaved mRNA levels by using qRT-PCR primers that span the miR159 binding site. Interestingly, *MYB33/MYB65* levels were much lower in both *MIR159a1* and *MIR159a2* lines compared to *mir159ab* ¸ but were not reduced to wild-type levels. Instead these intermediate *MYB* mRNA levels appeared dependent on the number of central mismatches, with more mismatches correlating with higher *MYB* mRNA levels (Figure 2A and B, Figure S4). This demonstrates that miR159a1 and miR159a2 can still reduce *MYB33/MYB65* transcript levels, suggesting that the miR159a variants are still mediating cleavage despite the existence of central mismatches.

**Figure 3:**
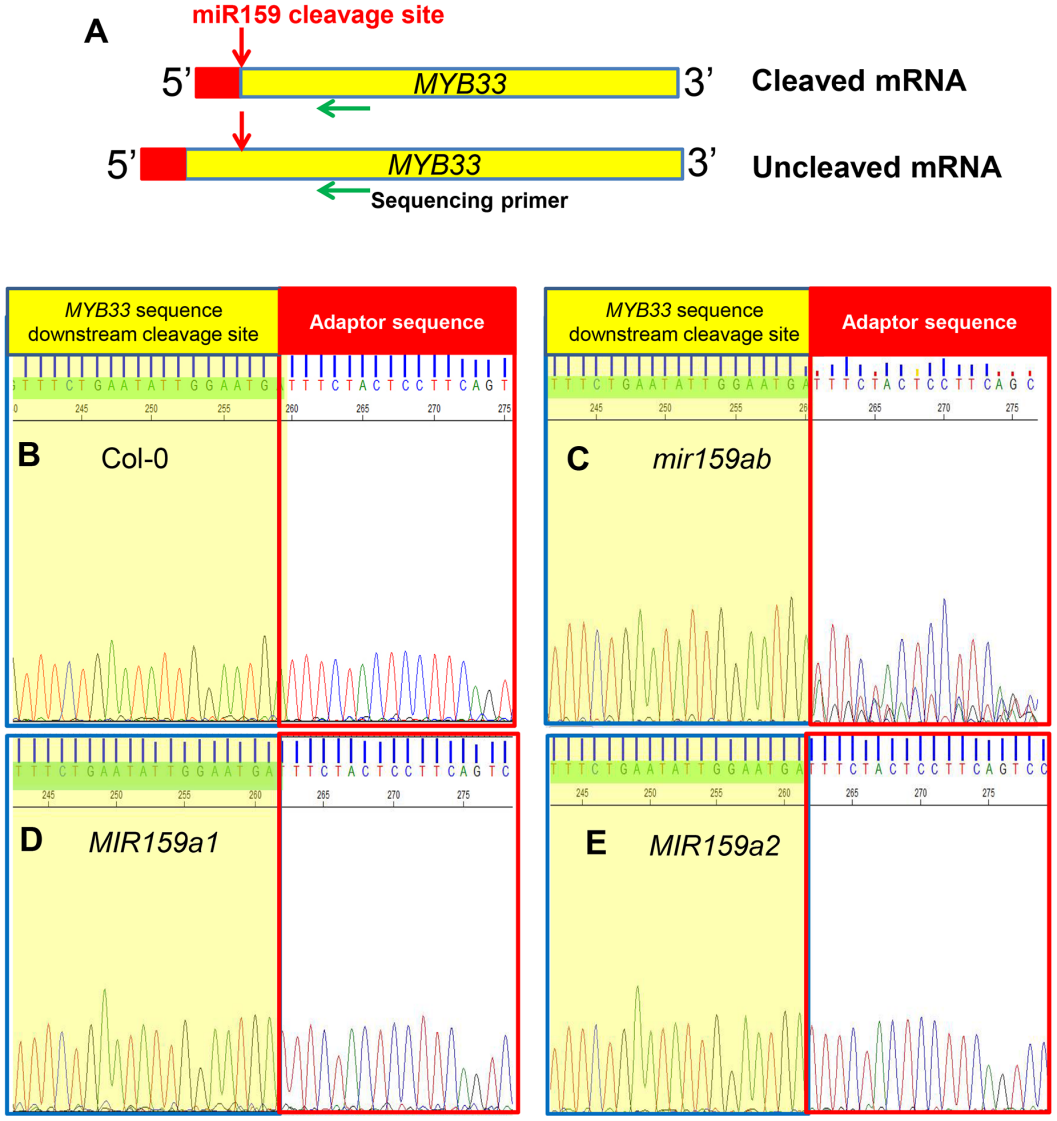
MiR159a variants with up to two central mismatches can direct target cleavage at the canonical miR159 cleavage site. (A) Schematic representation of *MYB33* PCR products after a modified 5′-RACE procedure to determine the proportion of degraded *MYB33* mRNA that corresponds to miR159-guided cleavage products. Red box: RNA Oligo adaptor; Yellow box: *MYB33* sequences; Red arrow: canonical miR159 cleavage site; Green arrow: *MYB33* sequencing primer. (B–E) Sequencing chromatographs of the cDNA-adaptor region from 5′-RACE recovered 3′ *MYB33* transcripts in: (B) Col-0, (C) *mir159ab*, (D) *MIR159a1* and (E) *MIR159a2* plants. Peaks from *MYB33* sequence downstream of the miR159 cleavage sites are highlighted in yellow, while those from the adaptor are left white. Three independent transgenic lines were checked for each construct and the results were identical.

To investigate this possibility, we performed a modified 5′-RACE miRNA cleavage assay [26]–[28]. Instead of gel purifying and cloning PCR products with sizes similar to the expected cleavage product followed by sequencing individual clones, we directly sequenced the entire PCR reaction and analysed the chromatograph peaks to approximate the proportion of degraded *MYB33* transcripts that correspond to miR159-guided cleavage products (Figure 3A and S3). For the wild-type control sample, sequencing of the 5′-RACE reaction using a *MYB33*-specific primer resulted in clean single peaks with very little baseline noise for each *MYB33* nucleotide up to the miR159-guided cleavage site, and then into the RNA adaptor sequences (Figure 3B). Such a clean signal indicates that the vast majority of degraded *MYB33* transcripts amplified by this assay were miR159-guided 3′-end cleavage products, which is consistent with previous strong degradome signatures of *MYB33* [8], [9]. By contrast, the *mir159ab* sample exhibited overlapping peaks in the portion of the sequence immediately upstream of the miR159 cleavage site (Figure 3C). This demonstrates that the degraded *MYB33* transcripts recovered by 5′-RACE in *mir159ab* were no longer predominantly miR159-guided cleavage products, but mixed populations arising independently of miR159-guided cleavage. By contrast, the chromatographic traces of reactions from *MIR159a1* and *MIR159a2* plants were indistinguishable from that of wild type (Figure 3D and 3E). This clearly demonstrates that miR159a1 and miR159a2 are capable of mediating cleavage despite the presence of central mismatches. Additionally, as this cleavage is occurring at the canonical *MYB33* cleavage site, this suggests that that the intended miR159a1 and miR159a2 variants are being processed from the modified *pri-MIR159a* transcripts, rather than other possible products arising from imprecise *DCL1* processing.

Although these 5′-RACE assays demonstrate that miR159a1 and miR159a2 can guide cleavage of *MYB33*, these assays are not quantitative. Therefore, a qRT-PCR assay was developed to quantitate the amount of miR159-guided 3′-end cleavage products of *MYB33* mRNA. An equal amount of total RNA from each sample was ligated to a 5′-end RNA adaptor followed by retro-transcription with poly T primers. To specifically amplify miR159-guided cleavage products, a hybrid forward primer was designed that included the last 19 nucleotides of the adaptor followed by five nucleotides of *MYB33* sequence immediately downstream of the miR159 cleavage site (Figure S5). The specificity of the assay was confirmed by testing Col-0 and *mir159ab* samples with two different forward primers (Figure S5). Both un-cleaved *MYB33* mRNA and 3′ end cleavage products were measured and the ratio was calculated to obtain an indication of miR159 cleavage efficiency, where the higher this ratio is the more inefficient cleavage is.

**Figure 4:**
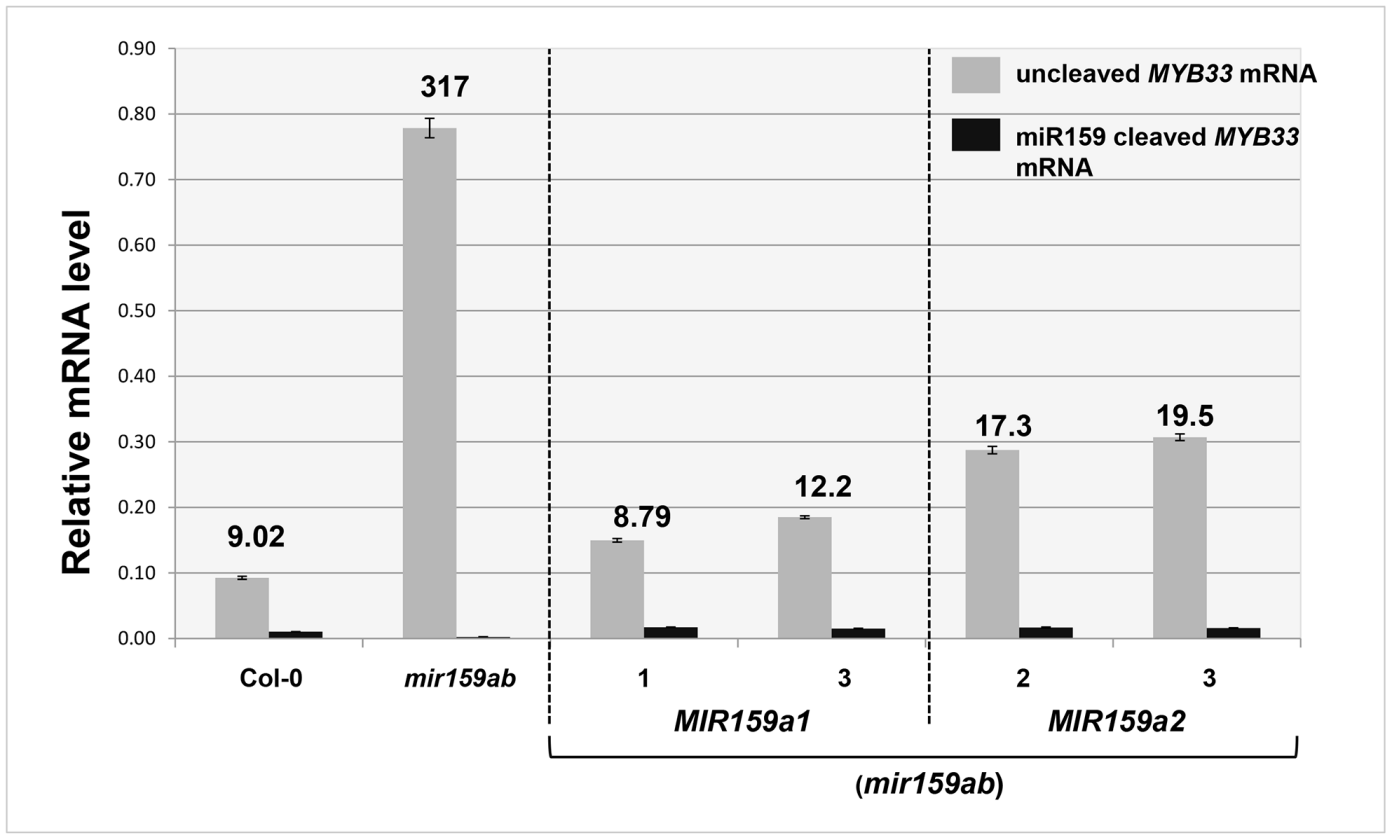
Quantitative comparison of un-cleaved *MYB33* mRNA and miR159-guided 3′-end *MYB33* cleavage products in *MIR159a1* and *MIR159a2* variants. Grey bars represent the un-cleaved *MYB33* transcript levels in Col-0, *mir159ab*, and independent transgenic lines of *MIR159a1* (*mir159ab*) and *MIR159a2* (*mir159ab*). Black bars represent the levels of miR159-guided 3′-end *MYB33* cleavage products in the same lines. Numbers show the relative ratio of un-cleaved transcripts to cleavage products. All mRNA levels were normalized with *CYCLOPHILIN 5*. Measurements are the average of three technical replicates. Similar results were obtained with two independent biological replicates. Error bars represent the SEM.

For wild-type, the steady levels of the miR159-guided 3′end *MYB33* cleavage products were detectable, but were approximately 9-fold lower than the un-cleaved *MYB33* mRNA levels (Figure 4). Such a low amount could be considered consistent with rapid degradation of these products by exoribonuclease XRN4 [9], [29]. For *mir159ab*, the levels of 3′ end cleavage products were negligible (Figure 4). Supporting the notion that the miR159a variants are able to guide cleavage, independent transgenic *MIR159a1* plants had comparable ratios to wild type, however the ratio values approximately doubled in *MIR159a2* plants. Together, these data suggest whilst miR159a2 can still direct cleavage, but it has been attenuated by the introduction of two central mismatches (Figure 4).

### MiR159 can strongly suppress high levels of un-cleaved *MYB33* transcripts

**Figure 5:**
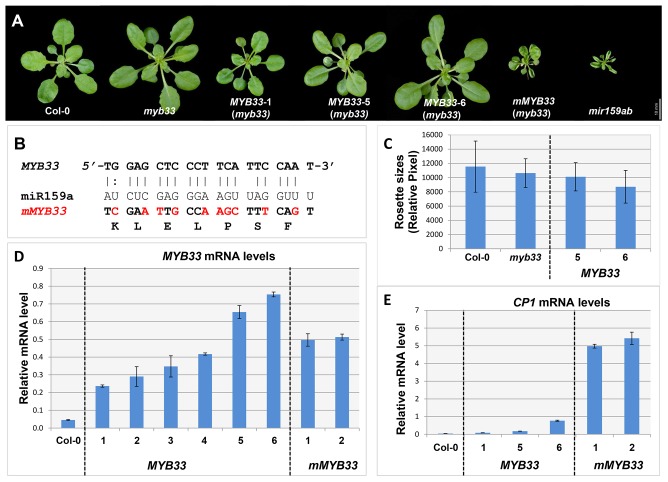
High levels of un-cleaved *MYB33* transcripts remain strongly repressed. (A) Aerial view of 29-day-old rosettes from *MYB33* and *mMYB33* transgenic plants (*myb33* genotype) grown under short day conditions. Scale bar = 10 mm. (B) Alignments between miR159a and the wild type and mutated miR159 target sites in *MYB33* and *mMYB33* constructs used to transform *Arabidopsis*. Nucleotide changes made in *mMYB33* are highlighted in red. Both *MYB33* and *mMYB33* encode the same protein. (C) Average rosette sizes of 29-day-old T2 transgenic plants from *MYB33* lines grown side by side with Col-0 and *myb33*. Unit of measurement shown is relative pixel. Multiple plants for each line were grown on soil side by side with Col-0 and *myb33*. More than fifteen transgenic plants in each line were analysed. Error bars represent SD. (D) The steady state mRNA levels of un-cleaved *MYB33* in the rosettes of multiple *MYB33* (lines 1–6) and *mMYB33* (lines 1–2) T2 transgenic lines. (E) Relative mRNA levels of *CP1* in the rosettes of the same *MYB33* and *mMYB33* lines. All mRNA levels were normalized with *CYCLOPHILIN 5*. All measurements are the average of three technical replicates with error bars representing the SEM.

Despite the higher levels of un-cleaved *MYB33* and *MYB65* transcripts, *MIR159a1* and *MIR159a2* plants appeared morphologically indistinguishable from wild type and the *CP1* mRNA remained essentially at wild type levels (Figure 1D). This leads to the question of whether miR159 also recruits a non-cleavage mechanism to achieve complete silencing of *MYB33* and *MYB65*, especially when cleavage is attenuated. To investigate this, we attempted to generate transgenic plants that strongly transcribe *MYB33*. To do this, *MYB33* or *mMYB33* (miR159-resistant) constructs were generated (Figure 5B) using a genomic *MYB33* clone [30] which contains the endogenous *MYB33* promoter. These constructs were transformed into the loss-of-function *myb33* mutant, a T-DNA knock-out allele of the *MYB33* gene [30]. *Myb33* is phenotypically indistinguishable from wild type and the absence of endogenous *MYB33* transcript enables accurate quantitation of the *MYB33* and *mMYB33* transgene mRNA levels.

Consistent with previous experiments [30], [31], expression of *mMYB33* caused pleiotropic developmental defects in *myb33*, as all 20 primary transformants had upwardly curled leaves and dwarfed stature resembling *mir159ab* (Figure 5A). In stark contrast, none of the 30 *MYB33* primary transformants recovered had any conspicuous defects (Figure 5A). QRT-PCR was then performed on rosettes to quantitate the steady state level of un-cleaved *MYB33* transcripts. As would be predicted, the *MYB33* transcript level was elevated in *mMYB33* lines (1 and 2), being approximately ten fold higher compared to wild type (Figure 5D). Surprisingly, all six *MYB33* transgenic lines examined also accumulated un-cleaved *MYB33* transcripts to a much greater level than wild type, where 5–15 fold increases were observed (Figure 5D; lines 1–6). This suggests that miR159 is unable to reduce *MYB33* mRNA to a wild type level through a transcript cleavage mechanism. Moreover, in *MYB33* lines 5 and 6, the un-cleaved *MYB33* mRNA levels were even higher than those in the *mMYB33* lines. The expression of these transcripts appeared to be strongly repressed by miR159, as the corresponding transgenic plants exhibited no curly leaves or reduction in rosette size (Figure 5A and 5C). In addition, whereas the *CP1* mRNA level was dramatically elevated in *mMYB33* plants (∼120 times higher than wild type), such increases were not seen in any *MYB33* plants (Figure 5E). These data together support the previous notion that miR159 suppresses *MYB33* not only by transcript cleavage, but also by a non-cleavage mechanism(s) [22]. Therefore, in this case, the steady state mRNA levels are a poor indicator of silencing.

**Figure 6:**
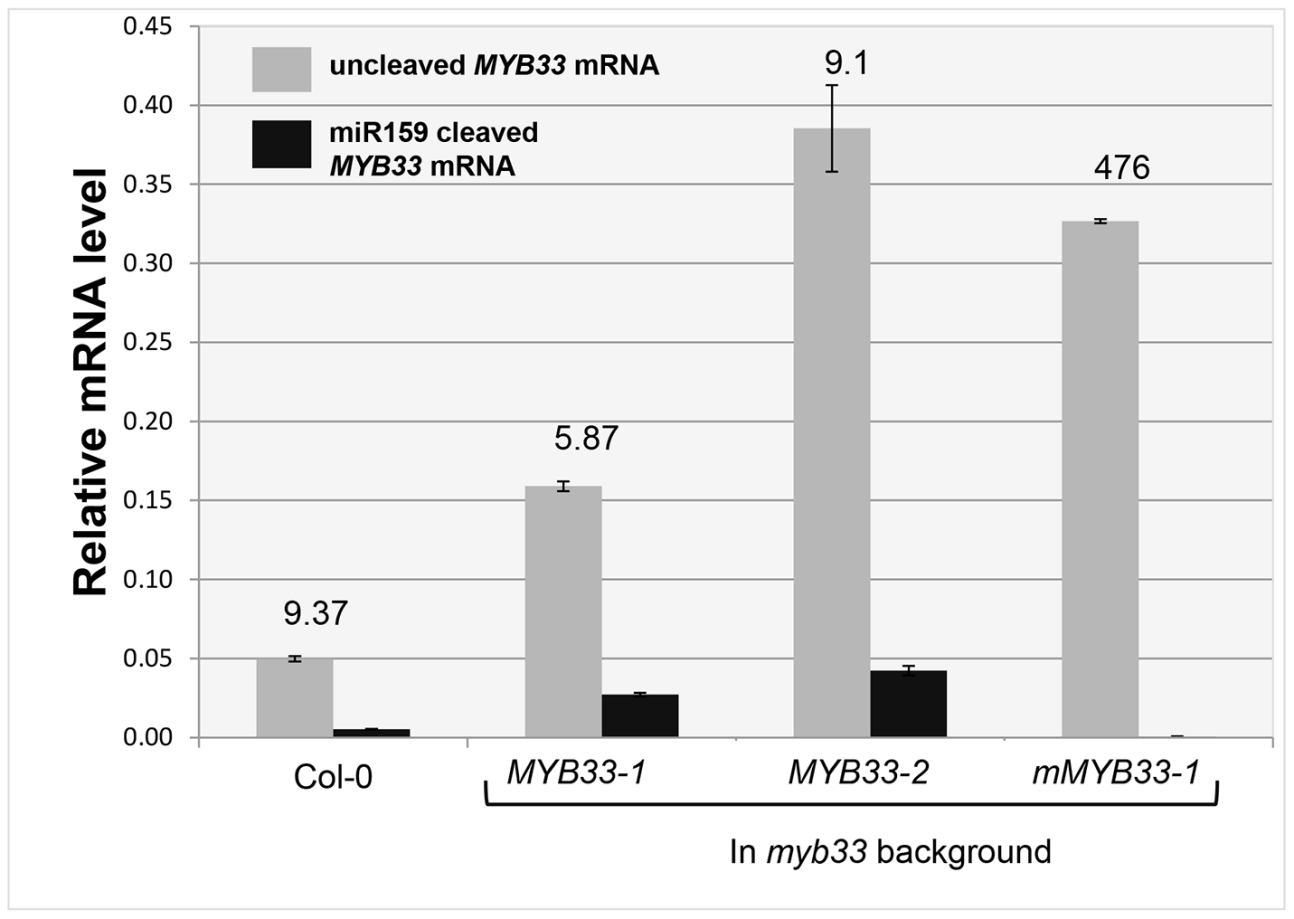
Quantitative comparison of un-cleaved *MYB33* mRNA and miR159-guided 3′-end *MYB33* cleavage products in *MYB33* and *mMYB33* plants. Grey bars represent the un-cleaved *MYB33* transcript levels in Col-0, *MYB33* and *mMYB33* transgenic lines. Black bars represent the levels of miR159-guided 3′-end *MYB33* cleavage products in the same lines. Numbers show the relative ratio of un-cleaved transcripts to cleavage products. All mRNA levels were normalized with *CYCLOPHILIN 5*. Measurements are the average of three technical replicates. Similar results were obtained with independent biological replicates. Error bars represent the SEM.

To investigate the reason why miR159 fails to repress *MYB33* mRNA to wild type levels in *MYB33* plants, we quantified 3′-end *MYB33* cleavage products and un-cleaved *MYB33* transcript levels as previously described. Interestingly, in the *MYB33* samples (lines 1 and 2), the 3′ end cleavage product appeared to increase proportionally with the levels of uncleaved *MYB33* mRNA, while no 3′ end cleavage product was apparent in the *mMYB33* sample (Figure 6). This indicates that cleavage is unlikely to be attenuated by the high *MYB33* transcript level, and the amount of cleavage products present appears dependent on the amount of *MYB33* transcripts present.

### *MYB33* transgenes with central mismatches cannot be fully silenced by endogenous miR159

**Figure 7:**
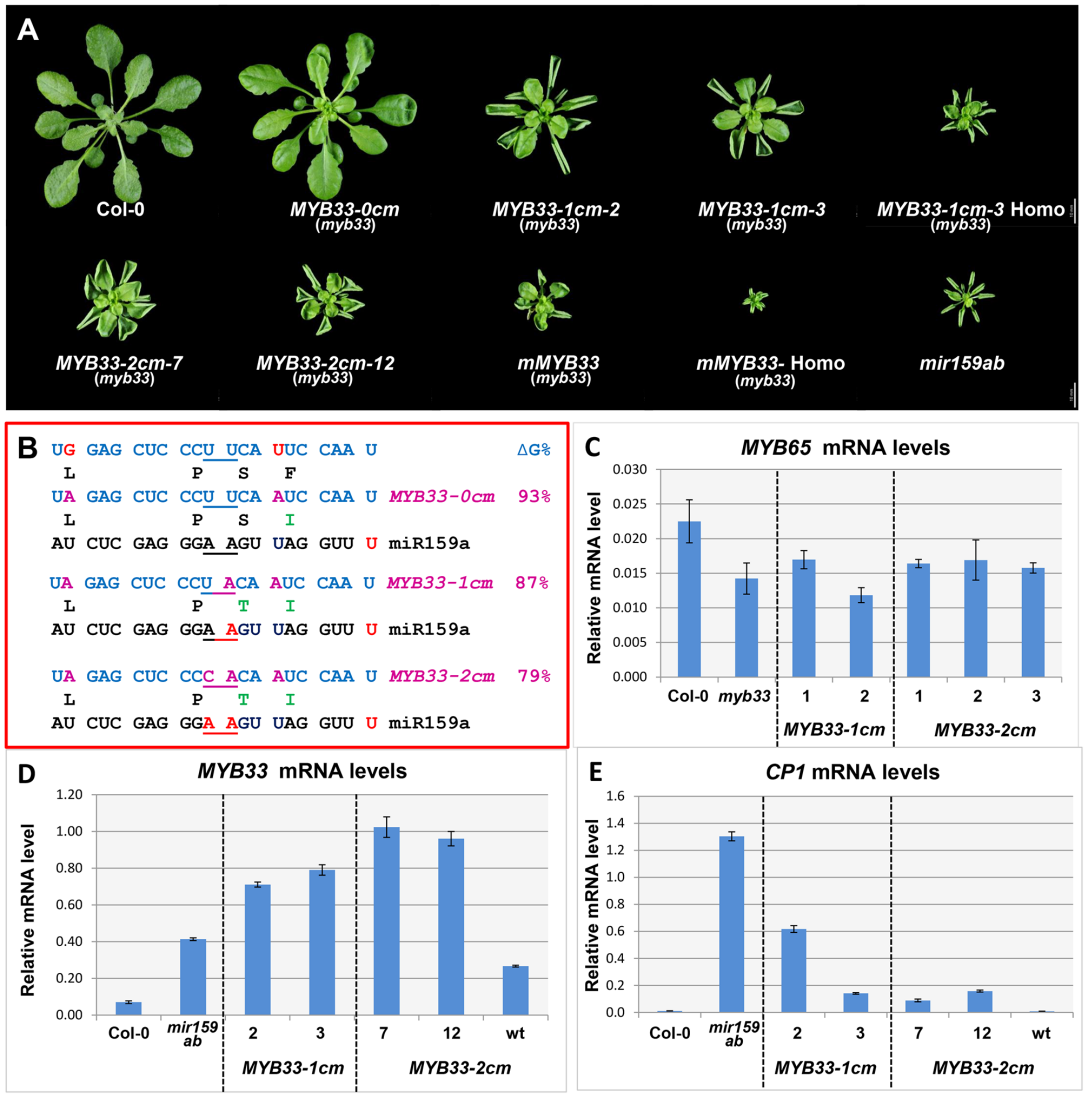
*MYB33* variants with central mismatches are not fully silenced. (A) Aerial view of rosettes from 35-day-old *MYB33-1 cm* and *MYB33-2 cm* plants (*myb33* genotype) grown side by side with Col-0, *mir159ab* and *mMYB33* under short day conditions. Homo stands for homozygous plants for the transgene. Scale bar = 10 mm. (B) The alignment between the different *MYB33* variants and miR159a. The sequences of the amino acids encoded by the miR159 target site in different *MYB33* transgenes are shown below. The percentage of free energy when pairing to miR159a compared to the perfect match is shown on the right. Red: mismatches; Purple: nucleotides mutated in the *MYB33* miR159 target site. Amino acid changes compared to the original MYB33 protein are highlighted with green. (C) The steady state *MYB65* mRNA level in the rosette of selected *MYB33-1 cm* and *MYB33-2 cm* transgenic lines with mutant phenotypes. Levels are not statistically different between the variants and parental *myb33* plants (D) The steady-state *MYB33* mRNA levels in rosettes of the transgenic lines shown in (A). (E) *CP1* mRNA levels in the rosette of the transgenic lines shown in (A). All mRNA levels were normalized with *CYCLOPHILIN 5*. Measurements are the average of three technical replicates. Error bars represent the SEM.

The miR159a variants result could lead to a surprising conclusion that central matches are not important for a strong silencing outcome. To further investigate this, three *MYB33* variants were made, all of which were identical except that the number of mismatches to miR159 at positions 10 and 11 ranged from zero to two (Figure 7B). To keep the overall complementarity and free energy close to the original value when pairing to miR159a, the original mismatches at positions 7 and 20 were corrected (Figure 7B). Therefore, the positions of mismatches of *MYB33-0 cm*, *MYB33-1 cm* and *MYB33-2 cm* to miR159a mirror those of miR159a0, miR159a1 and miR159a2 to the endogenous *MYB33/MYB65* genes respectively. Although these sequence alterations result in one to two amino acid changes in the MYB33 protein, all substitutions made were conservative changes to minimize possible changes to the biochemical properties of the protein. Primary transformants for each construct were generated in the *myb33* background.

Consistent with the result that *mir159ab* can be fully complemented by miR159a0, all 52 *MYB33-0 cm* transgenic plants generated were indistinguishable from wild type (Figure 5A). However, counter to miR159a1 and miR159a2, all 46 *MYB33-1 cm* and 41 out of 42 *MYB33-2 cm* T1 plants recovered had developmental defects, exhibiting phenotypes characteristic of *mir159ab*, including curled leaves and a smaller rosette (Figure 7A). This demonstrates that the *MYB33-1 cm* and *MYB33-2 cm* transgenes are not efficiently silenced by endogenous miR159. Molecular and phenotypic analyses were carried out to investigate this. Firstly, to dismiss that *MYB33-1 cm* and *MYB33-2 cm* are acting as decoys to suppress endogenous miR159 activity [32], we measured *MYB65* transcript levels in the *MYB33* variants and control plants. As the mRNA levels were found to be similar in all plants (Figure 7C), this demonstrates that *MYB65* has not been deregulated in *MYB33* variants, meaning that miR159 activity has not been perturbed. Therefore, the abnormal rosette phenotypes were resulting from failure of miR159 to silence the *MYB33* variants. To phenotypically assess the severity of the developmental phenotypes, multiple *MYB33-1 cm* and *MYB33-2 cm* T2 transgenic lines were grown side by side with Col-0, *mir159ab* and *mMYB33* plants. Although most transgenic *MYB33-1 cm* and *MYB33-2 cm* lines displayed traits characteristic of *mir159ab*, the extent of leaf curl and the reduction in rosette size of most plants were less severe compared to either *mir159ab* or *mMYB33* plants (Figure 7A). Therefore, the repression of *MYB33-1 cm* or *MYB33-2 cm* by miR159 does not appear completely abolished.

To examine this, total RNA was extracted from individual transgenic lines using rosettes of multiple plants exhibiting similar phenotypes (Figure 7A). QRT-PCR analysis found that the un-cleaved *MYB33* mRNA levels were 10–15 fold higher in *MYB33-1 cm* and *MYB33-2 cm* lines compared to wild type, while only a six-fold increase was observed in *mir159ab* (Figure 7D). Even considering the fact that *MYB65* is also de-regulated in *mir159ab*, these very high *MYB33-1 cm/2 cm* mRNA levels would have been expected to result in phenotypic severities more akin to that of *mir159ab*, if they were completely devoid of miR159 regulation. Moreover, transcript levels of *CP1*, which reflects the abundance of MYB protein, were found to be much lower in all *MYB33-1 cm/2 cm* lines examined than in *mir159ab* (Figure 7E). Together, these data suggest that the high levels of *MYB33-1 cm* and *MYB33-2 cm* transcripts are still being partially repressed by miR159, which again suggests a non-cleavage mechanism in operation.

**Figure 8:**
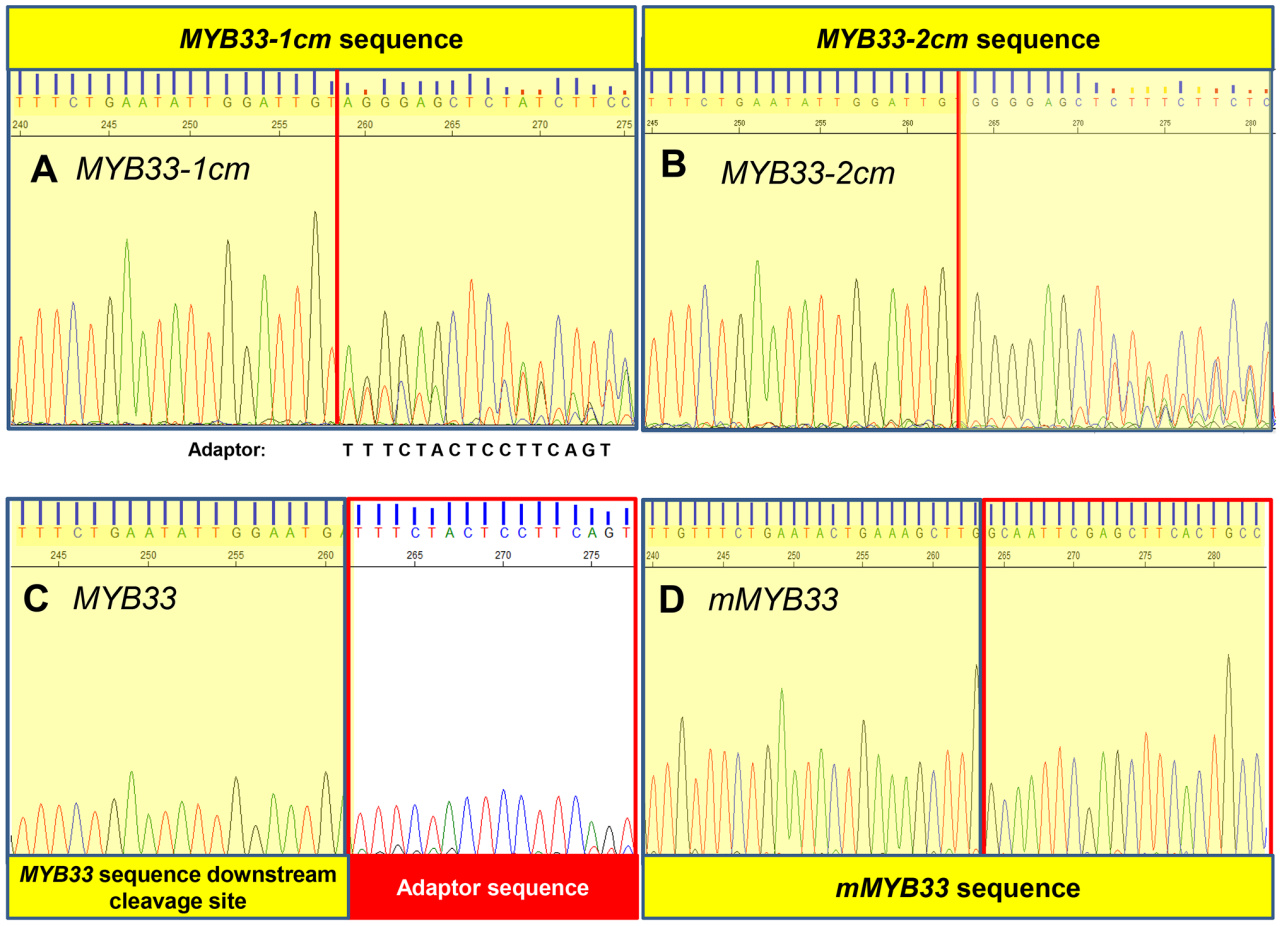
MiR159-guided cleavage is attenuated in *MYB33-1 cm* and *MYB33-2 cm* plants. A fragment of the sequencing chromatography from 5′-RACE recovered 3′ *MYB33* transcripts in (A) *MYB33-1 cm* plants. (B) *MYB33-2 cm* plants. (C) *MYB33* plants. (D) *mMYB33* plants. *MYB33-1 cm*, *MYB33-2 cm*, *MYB33* and *mMYB33* sequences are highlighted yellow, while the adaptor is left white. Red line: canonical miR159 cleavage site.

5′-RACE was also performed to assay the degraded 3′-end *MYB33* mRNA population as described before (Figure S3). A *MYB33* transgenic line with a very high *MYB33* mRNA level was included as control (line 5 from Figure 7), and its sequencing chromatograph was identical to that of wild type (Figure 8C). This demonstrates that the vast majority of 3′ degraded *MYB33* transcripts amplified by this assay are miR159-guided 3′ end cleavage products, regardless of how high the transcript level is. In contrast, no signals from the adaptor sequence but those from the transgene were recovered for *mMYB33*, which is completely resistant to cleavage (Figure 8D). For *MYB33-1 cm*, past the cleavage site, the signal became a mixture of adaptor and *MYB33-1 cm* sequence, indicating that although some miR159-guided cleavage products were present, it was a mixed population (Figure 8A). For *MYB33-2 cm*, the signal from the adapter sequence corresponding to the canonical miR159 cleavage site was negligible, where the signal was dominated by *MYB33-2 cm* sequences (Figure 8B). This demonstrates that 3′ end miR159-guided cleavage products are poorly represented in the reactions from the *MYB33-1 cm* and *MYB33-2 cm* samples when compared to the *MYB33* sample, arguing that miR159-guided cleavage of *MYB33-1 cm* and *MYB33-2 cm* is not as efficient as *MYB33*, which is likely due to the introduction of central mismatches.

### MiRNA:target stoichiometry has a critical impact on the silencing outcome in *MIR159a* and *MYB33* variant plants

It appears that central mismatches attenuate cleavage in both the miR159a variant-*MYB33*/*MYB65* and miR159-*MYB33* variant relationships, therefore additional factors must be impacting the differential silencing outcomes observed. Firstly, although the duplex pairs have identical mismatch positions, the nucleotides changes are not identical and hence will result in duplexes with different thermodynamics stabilities (Table S1), possibly impacting the silencing outcome. Secondly, although *MYB33* and *MYB65* were being transcribed at wild type levels in *MIR159a1* and *MIR159a2* plants, it is clear that mRNA from *MYB33*, *MYB33-1 cm* or *MYB33-2 cm* transgenes accumulated to higher levels than endogenous *MYB33* (Figure 5D, 7D). This would be predicted to alter the stoichiometric ratio of *MYB33* transcripts to miR159. Since target mRNA: miRNA stoichiometry is important for the silencing outcome in animal miRNA-target genes relationships [33]–[35], an altered miR159: *MYB33* stoichiometry may explain the observed differential silencing outcomes.

As it is impossible to introduce identical central mismatch nucleotides in both the miR159a variant-*MYB* and miR159-*MYB33* variant duplexes, we aimed to alter the stoichiometric ratio between *MYB33* and *miR15*9 in the *MIR159a* and *MYB33* variant plants instead. This will determine whether stoichiometry can alter the silencing outcome, or whether the different thermodynamic stabilities of two different duplexes override any stoichiometric alteration.

**Figure 9:**
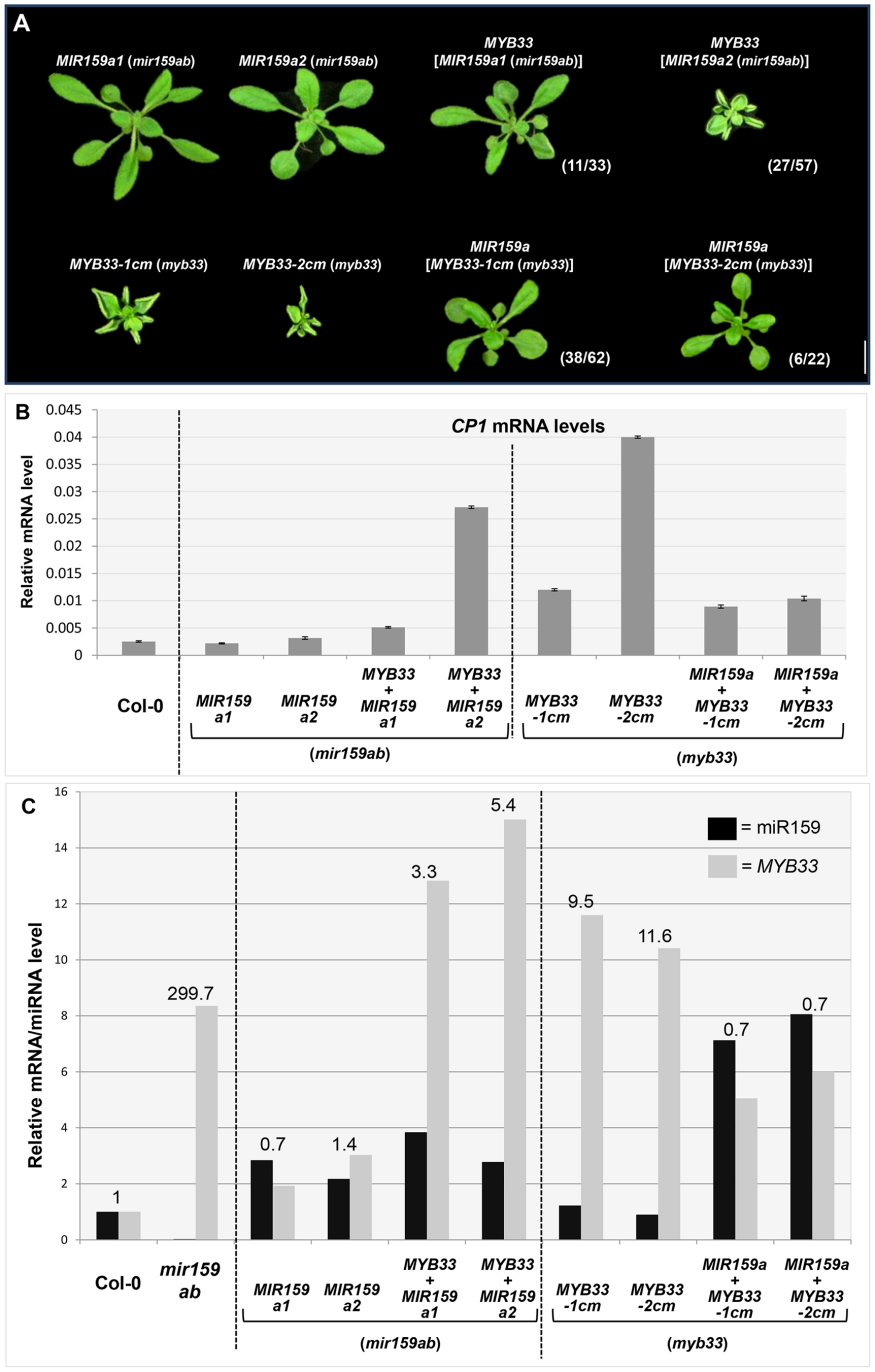
*MYB33*:miR159a stoichiometry has a critical impact on the silencing outcome in *MIR159a* and *MYB33* variants. (A) Aerial view of rosettes of 27-day-old of *MIR159a1* (*mir159ab*) and *MIR159a2* (*mir159ab*) plants transformed with the *MYB33* construct; and *MYB33-1 cm* (*myb33*) and *MYB33-2 cm* (*myb33*) plants transformed with *MIR159a*. All plants were grown side by side with the original *MIR159a1*, *MIR159a2*, *MYB33-1 cm* and *MYB33-2 cm* parental lines used for transformation. Scale bar = 10 mm. (B) The mRNA levels of *CP1* in transgenic lines shown in (A). (C) The relative un-cleaved *MYB33* mRNA level (grey bars) and mature miR159a/variant levels (black bars) in Col-0, *mir159ab* and transgenic lines shown in (A). All original mRNA levels were normalized with *CYCLOPHILIN 5*. All original miR159a levels were normalized with *snoR101*. All fold changes in (C) are relative to the levels in Col-0, which is set as 1. Measurements are the average of three technical replicates. Error bars represent the SEM.

Firstly, the wild type *MYB33* transgene was transformed into individual *MIR159a1* and *MIR159a2* T3 transgenic lines (*mir159ab* genotype, wild-type phenotype) to see whether higher levels of *MYB33* transcript could override the complementation by the miR159a variants. In contrast to the introduction of *MYB33* into wild-type plants that resulted in no phenotypic abnormalities (Figure 5), the introduction of the *MYB33* transgene into multiple lines of *MIR159a1* and *MIR159a2* plants resulted in a large proportion of transgenic plants displaying the characteristic phenotypic abnormalities of *MYB33* expression, including leaf curl and smaller rosette size (Figure 9A). QRT-PCR analysis confirmed strong increases of *MYB33* transcript levels in these *MYB33* [*MIR159a1* (*mir159ab*)] and *MYB33* [*MIR159a2* (*mir159ab*)] plants, whilst the mature miR159a1 and miR159a2 levels remained similar (Figure 9C). Consistent with the morphological defects, *CP1* mRNA levels were elevated, especially in the *MYB33* [*MIR159a2* (*mir159ab*)] plants. This clearly demonstrates that the *MIR159a* variants are poor silencers of *MYB33* when *MYB33* is highly transcribed, resulting in an unfavourable stoichiometric miR159:*MYB* ratio for silencing.

Secondly, the wild-type *MIR159a* construct was transformed into *MYB33-1 cm* and *MYB33-2 cm* T3 transgenic lines. The severe leaf curl and small rosette size observed in *MYB33-1 cm* and *MYB33-2 cm* plants were largely suppressed in many *MIR159a* (*MYB33-1 cm*) and *MIR159a* (*MYB33-2 cm*) transformants (Figure 9A). The introduction of *MIR159a* resulted in elevated levels of mature miR159a by approximately five-fold and a simultaneous reduction of the *MYB33* transcript levels to approximately 50% of that found in parental *MYB33-1 cm* and *MYB33-2 cm* lines (Figure 9C). Consistent with these observations, the levels of *CP1* were suppressed in these *MIR159a* (*MYB33-1 cm*) and *MIR159a* (*MYB33-2 cm*) transformants (Figure 9B).

A ratio between the relative abundance of *MYB33* and miR159a was calculated (levels of both *MYB33* and miR159a in Col-0 were normalized to 1, Figure 9C). The higher this ratio was, the poorer the silencing outcome became; and vice versa. These data clearly demonstrate that the miRNA: target mRNA stoichiometric ratio has a critical role in determining the silencing outcome when central complementarity is compromised. Although we are unable to address whether thermodynamic differences between the duplexes may in part contribute to the differential silencing outcome, it, or any other factor, is clearly not strong enough to override stoichiometry.

### Conserved nucleotides flanking the miR159 binding site of *MYB33* are critical for efficient silencing

**Figure 10:**
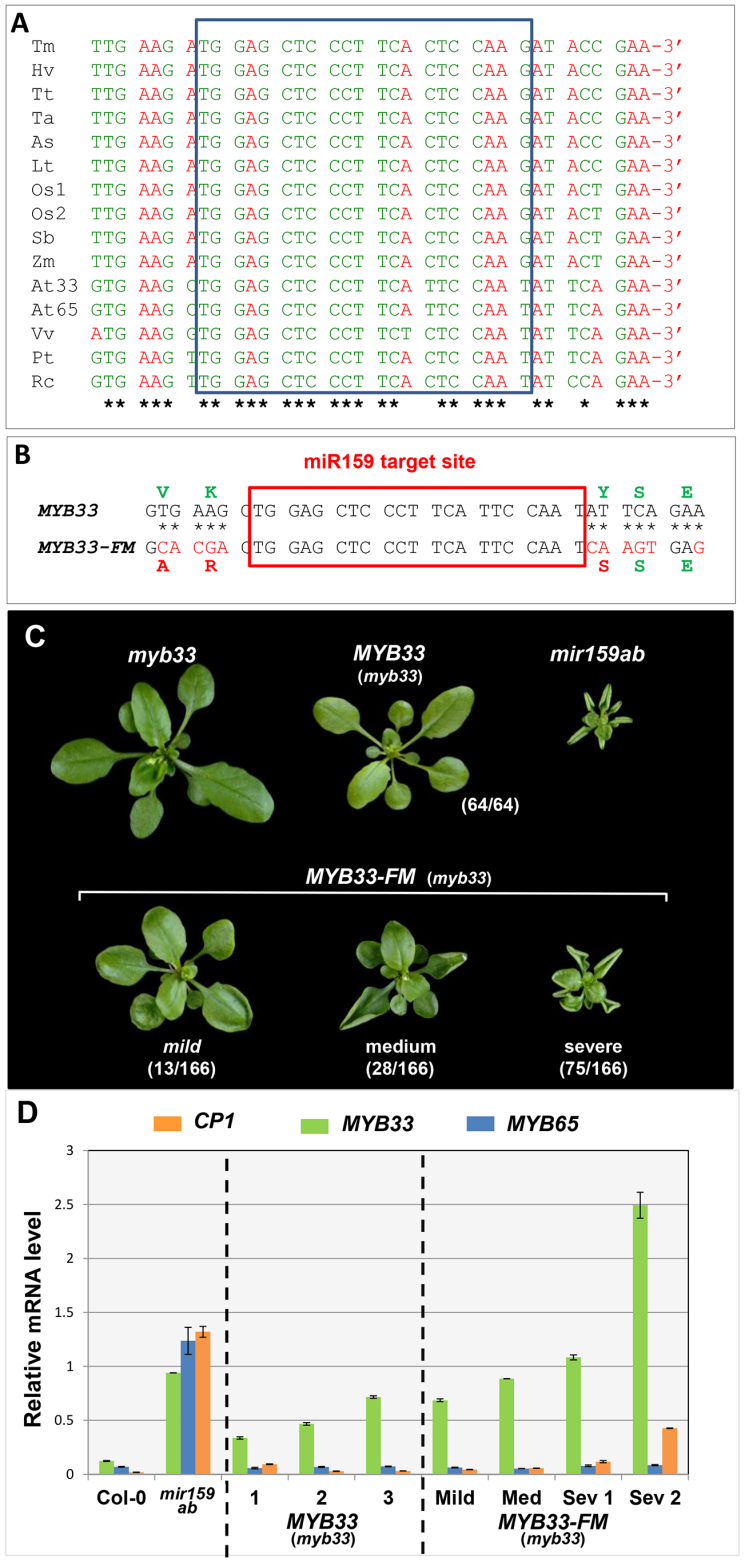
Nucleotides flanking the *MYB33* miR159 target site are critical for conferring efficient silencing. (A) ClustalW2 sequence alignment of *MYB33* homologues. Nucleotides are grouped into codons of the reading frame. Conserved nucleotides are marked with *. The miR159 binding site is indicated with a pale blue box. Sequences include genes from monocotyledonous species (Tm: *Triticum monococcum*, Hv: *Hordeum vulgare*, Tt: *Triticum turqidum*, Ta: *Triticum aestivum*, As: *Avena sativa*, Lt: *Lolium tremulentum*, Os: *Oryza sativa*, Sb: *Sorghum bicolor*, Zm: *Zea mays*) and dicotyledonous species (At: *Arabidopsis thaliana*, Vv: *Vitis vinifera*, Pt: *Populus trichocarpa*, Rc: *Ricinus communis*). (B) Mutations made in the regions flanking the miR159 target site in the *MYB33-FM* construct. Corresponding nucleotide and protein changes are denoted red. (C) Aerial view of 4-week-old *MYB33-FM* transgenic plants in comparison with *MYB33*, *myb33* and *mir159ab* plants grown side by side under long day conditions. The number of primary transformants showing the arbitrary phenotypic classification described in the text is listed in the bracket. Scale bar = 10 mm. (D) The mRNA levels of *MYB33*, *MYB65* and *CP1* in multiple biological samples of independent *MYB33* and *MYB33-FM* transformants. Mild, Med (medium) and Sev (severe) denote the strength of phenotypic abnormalities observed in *MYB33-FM* transformants sampled. All mRNA levels were normalized to *CYCLOPHILIN 5*. Measurements are the average of three technical replicates with error bars representing the SEM.

Interestingly, the stoichiometric ratio of endogenous miR159 to *MYB33* appears less important, as even in transgenic plants transcribing *MYB33* many fold higher than in wild-type, *MYB33* appears to be strongly silenced (Figure 5, 6). This argues that the sensitivity of *MYB33* to miR159 regulation is strong enough to override an unfavourable stoichiometric ratio. In order to understand this sensitivity, we initiated a characterization of the *MYB33* region containing the miR159 binding site by aligning genes encoding *MYB33* homologs from different monocotyledonous and dicotyledonous plant species. Alignment of the nucleotide sequences of 15 of these genes revealed multiple conserved nucleotides flanking the miR159 binding site, many of which are located in the third codon position (Figure 10A), suggesting that conservation is not at the protein but RNA level. Moreover, these nucleotides are among the strongest stretches of nucleotide conservation outside of the R2R3 MYB domain or the miR159 binding site (Figure S6), suggesting the conservation has functional significance.

To test their role in miR159 regulation of *MYB33*, eleven nucleotides flanking the miR159 binding site were mutated in the genomic *MYB33* construct to generate the *MYB33-Flanking site Mutant* construct (*MYB33-FM*, Figure 10B). These alterations result in sequence changes in the MYB33 protein, however all three resulting amino acid substitutions were conservative, minimizing possible changes to the biochemical properties of the protein. Moreover, these amino acids do not appear to be critical for *MYB33* function, as a previous study has shown that a 33 bp deletion in the *MYB33* coding region, including the miR159 binding site and these flanking nucleotides, still results in the production of a functional protein [30].

*MYB33-FM* and the positive control, *MYB33*, were individually transformed into *myb33* plants, and multiple primary transformants were selected and analyzed. Consistent with previous results, none of the *MYB33* plants showed any phenotypic abnormalities, indicating that *MYB33* is fully repressed by miR159 (Figure 10C). In contrast, 116 out of 126 *MYB33-FM* plants showed strong developmental defects characterized by curly leaves and stunted growth (Figure 10C). As these are phenotypic characteristics of *mir159ab*, it appears that miR159 regulation of *MYB33* has been compromised. Molecular analysis was carried out on RNA extracted from primary transformants that had been categorized according to phenotypic severities. As before, *MYB33* transcript levels were elevated in *MYB33* plants (Figure 10D), but remained strongly silenced as these plants were indistinguishable from wild-type (Figure 10D). Elevated *MYB33* levels were also observed in all *MYB33-FM* plants examined, however, in contrast to *MYB33* plants, the levels positively correlated with the phenotypic severity observed, as well as the *CP1* mRNA levels (Figure 10D). By contrast, *MYB65* mRNA levels were unchanged in all transgenic plants compared to wild type, indicating that miR159 activity has not been perturbed in any of the transgenic lines, therefore *MYB33-FM* is not acting as a decoy (Figure 10D). In conclusion, this data clearly demonstrates that in transgenic plants where *MYB33-FM* is highly transcribed, the silencing efficacy of miR159 against *MYB33-FM* is strongly perturbed.

### Flanking or central nucleotide mutations perturb miR159 efficacy to similar extents

Our data has shown that under a poor stoichiometric ratio, both the central and flanking nucleotides are critical for strong miR159 efficacy against endogenous *MYB33*. In an attempt to determine the degree to which *MYB33* silencing is perturbed by these different mutations, a *GUS*-reporter system was used to quantitatively measure silencing at both the mRNA and protein level. The *MYB33*, *MYB33-1 cm*, *MYB33-2 cm* and *MYB33-FM* transgenes were each translationally fused with a GUS reporter and corresponding transgenic lines were generated in *myb33* plants [30]. For each sample, more than 50 primary transformants were bulked together from which both RNA and protein samples were prepared, so that both transcript (qRT-PCR) and GUS activity (MUG assays) quantification could be then performed, enabling direct comparison of measurements.

**Figure 11:**
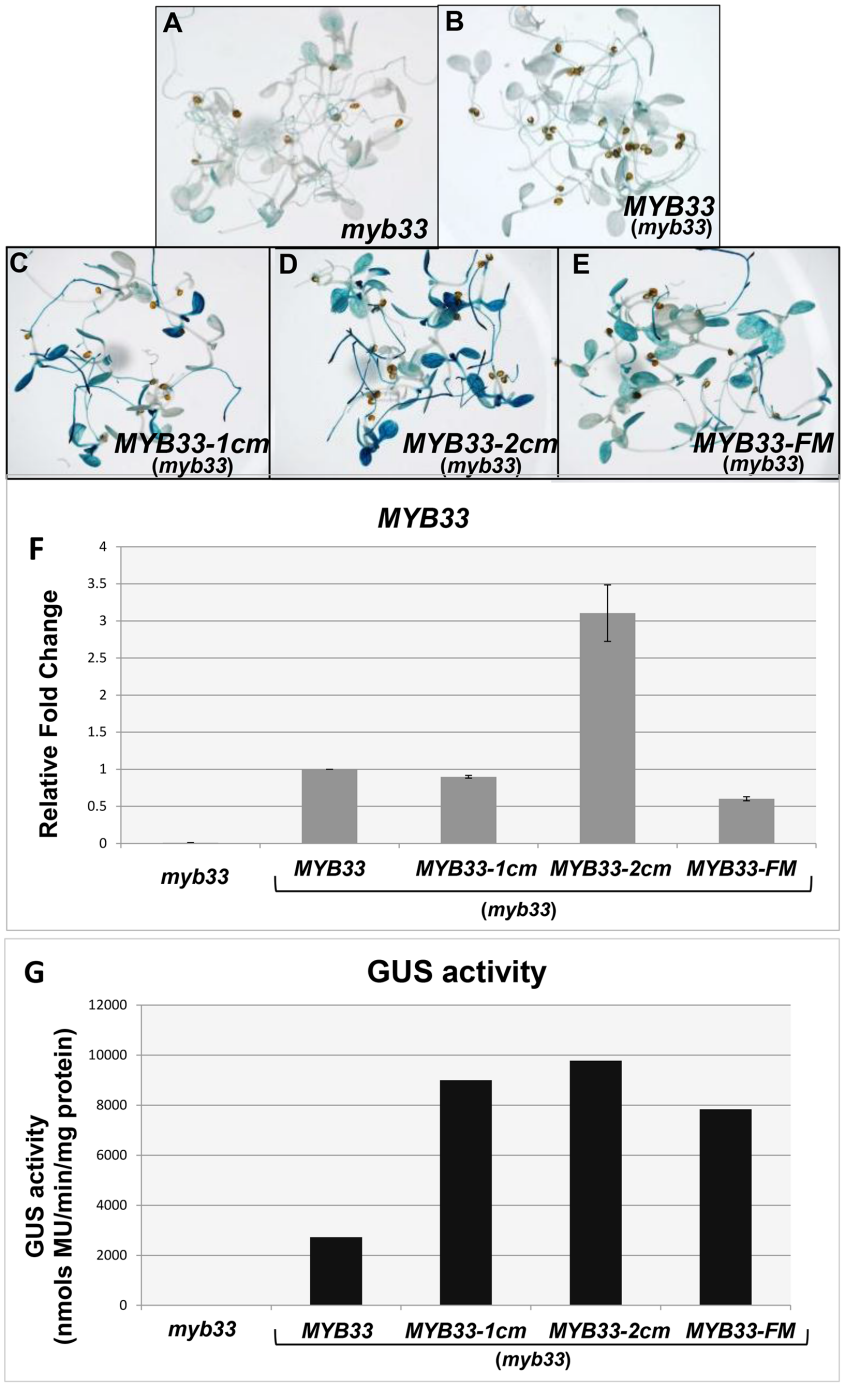
Quantification of *MYB33* expression using the GUS reporter system. *In situ* GUS staining of 8-day-old seedlings of the control genotype, (A) *myb33*, and independent transformants of (B) *MYB33:GUS*, (C) *MYB33-1 cm:GUS*, (D) *MYB33-2 cm:GUS*, (E) *MYB33-FM:GUS*; all in the *myb33* background. (F) The relative fold changes of *MYB33* mRNA levels. All values were averaged from two individual biological replicates and the level in *MYB33:GUS* was normalized to 1. (G) β-Glucuronidase (GUS) activity as measured by MUG assays. Measurements represent three technical replicates and error bars are too small to be visible.

Un-cleaved *MYB33* transcript levels in *MYB33-1 cm:GUS* and *MYB33-FM:GUS* were similar to the level in *MYB33:GUS* plants, whereas the *MYB33-2 cm:GUS* mRNA level was approximately three-fold higher (Figure 11F), again supporting that central mismatches attenuate cleavage. Despite *MYB33-1 cm:GUS* and *MYB33-FM:GUS* mRNA levels being similar to *MYB33:GUS*, these *MYB33-1 cm:GUS* and *MYB33-FM:GUS* mRNA must be translated more efficiently, as GUS activity in these lines are approximately three fold higher than in *MYB33:GUS* lines (Figure 11G). By contrast, the much higher *MYB33-2 cm:GUS* mRNA levels did not translate into dramatically higher GUS activity when compared to GUS activity in the *MYB33-1 cm:GUS* and *MYB33-FM:GUS* lines (Figure 11G), which again supports the existence of a strong non-cleavage silencing mechanism (Figure 2A).

Separately, *in situ* GUS staining was performed on over a hundred of primary transformants for each construct. The results were consistent with the MUG assays, with *MYB33-1 cm:GUS MYB33-2 cm*:GUS and *MYB33-FM-GUS* all having stronger staining than that of *MYB33:GUS* (Figure 11A–E). Therefore, the nucleotides that flank the miR159 binding site appear just as necessary for strong silencing of *MYB33* as the central nucleotides of its miR159 binding site. Together, these data argues that a miRNA “target” site encompasses not only the miRNA binding site, but the sequence context with which it is in.

## Discussion

Despite the extensive analysis of miRNAs in plants, there has been little investigation into the principles that govern their efficacy. Here, taking advantage of the miR159-*MYB33*/*MYB65* module as a model system, we have investigated the principles that control the efficacy of silencing by a highly conserved plant miRNA.

### Perfect central complementarity is not essential for strong miR159 efficacy

It is generally assumed that central matches are required for cleavage, and this is a prerequisite for a strong silencing outcome [36]. However, we functionally demonstrate *in planta* that miRNAs can potently silence target genes with two central mismatches. This directly challenges the empirical parameters of sequence requirements for miRNA-mediated gene silencing, which state there should be no mismatches at positions 10 and 11 of the miRNA-target gene pair [5]. There have been reports of single central mismatches at these positions in naturally occurring miRNA-target pairs. Firstly, degradome signatures have found such miRNAs that can cleave target mRNAs; however the strength of the silencing imposed or the functional outcome of these central mismatches is unknown [37]. Secondly, miR390 that can trigger tasiRNA production has a binding site which includes a single central mismatch, but it is unknown whether the miRNA can cleave its target *in vivo* [38].

However, it is clear that for strong regulation to occur under the scenario of central mismatches, the miRNA:target stoichiometric ratio must strongly favour the miRNA. Although we show that perfect central complementarity is not mandatory for miR159-guided cleavage, the introduction of central mismatches does attenuate cleavage. Firstly, the average steady state mRNA levels of *MYB33* and *MYB65* were elevated in *MIR159a1* and *MIR159a2* plants (Figure 3), where the ratio of un-cleaved *MYB33* transcripts to 3′ end cleavage products increased with the number of central mismatches (Figure 4). Secondly, the sequencing profiles of the 5′-RACE products from *MYB33-1 cm* and *MYB33-2 cm* plants imply that the cleavage of these centrally mismatched *MYB33* transgenes is attenuated (Figure 8). This is consistent with a large body of data that perfect central complementary promotes cleavage, whereas central mismatches compromise cleavage [3]. In fact, the *Arabidopsis* transcriptome has many mRNAs with potential miRNA binding sites with mismatches at positions 10 and 11, most of which have not been annotated as miRNA targets [32].

### A potent non-cleavage mechanism silences *MYB33*

As one of the best-studied canonical miRNA targets being cleaved by miR159 and characterised by a strong degradome signature [8], [9], here we show that *MYB33* is also efficiently silenced by a non-cleavage mechanism. In the case of *MYB33* (Figure 5) or *MIR159a2* (Figure 1–2) transgenic plants where *MYB33* mRNA levels are high, this mechanism becomes more apparent, where it suppresses *MYB33* expression to a phenotypically inconsequential level. This argues that cleavage is limiting to some degree, and this non-cleavage mechanism acts in combination to ensure silencing of un-cleaved transcripts. It is probable that this non-cleavage mechanism corresponds to mechanism commonly referred to as translational repression, the phenomenon where target protein accumulation is inconsistent with its transcript level due to miRNA regulation. The repression of *MYB33* at both the transcript and translational level is consistent with the proposal that most known plant miRNAs regulate their targets using both mechanisms [3], [18]. Indeed, most targets which are claimed to be regulated primarily at the translational level also undergo cleavage, where the compositions of the degradome signatures for these “translationally repressed” targets appear indistinguishable from miRNA targets that appear to be predominantly regulated by cleavage [8], [9]

It has been hypothesised that cleavage could be a mechanism that results in the fast targeted and irreversible clearing of regulatory mRNAs [14], which in terms of expression would result in a switch outcome [19]. Although miR159 totally silences *MYB33*/*MYB65*, it is obvious that miR159 does not clear *MYB33* transcripts, especially in the *MYB33* transgenic plants. However, miR159 repression of *MYB33* via this non-cleavage “translational repression” mechanism appears potent, where it seems to be having a “switch” effect. This is in contrast to “translational repression” in animals, which has been proposed to dampen or tune translation to obtain the desired level of protein synthesis [19].

### Factors beyond sequence complementarity: miRNA:target mRNA stoichiometry

Although sequence complementarity is generally the sole factor considered when predicting plant miRNA-mediated silencing, here we show there are other strong contributing factors for the silencing outcome. Firstly, we demonstrate that the silencing outcome can be strongly influenced by the stoichiometric ratio of miRNA to target, a factor that has been largely ignored in plants to date. By transgenically altering the relative abundance of either *MYB33* target or miR159 in the variant plants, we could change the silencing outcome independent of complementarity. It appears that the more inefficient a miRNA–target interaction is, the more important stoichiometry becomes. For instance, a combination of high target concentration together with mismatches at the cleavage site resulted in a poor silencing efficacy. This is consistent with previous findings in animals, where a target with central mismatches can saturate the miRNA at a lower concentration than a perfect matched target [33], [34]. This is because the miRISC catalytic rate is slowed down by central mismatches, therefore sequestering the miRNA from further rounds of silencing [35]. As miRISC recycling would make the silencing process more energy efficient, this could be a strong selective driver for perfect central matches in miRNA-target duplexes, which the vast majority of *bona fide* miRNA-target pairs contain [5].

### Factors beyond sequence complementarity: the sequence context of the miR159 binding site

We have clearly demonstrated that nucleotides flanking a miRNA binding site can impact the efficacy of miRNA-mediated silencing. Based on the facts that the vast majority of *MYB33-FM* plants had developmental defects and the GUS expression level of *MYB33-FM:GUS* was similar to *MYB33-2 cm*:GUS, these flanking nucleotides clearly have a large impact on the efficacy of miR159-mediated silencing. This provides the first evidence in plants that the sequence in which a miRNA binding site is embedded plays a critical role in miRNA-target interactions, possibly affecting the silencing outcome comparably to nucleotides within the binding site itself.

We speculate that these flanking nucleotides provide a favourable context for recognition of the *MYB33* mRNA by miR159, with important consequences for the efficacy and specificity of their interaction. We have previously shown that miR159 is functionally specific for *MYB33* and *MYB65* despite bioinformatics predicting approximately 20 genes to be miR159 regulated in *Arabidopsis* [21]. It is possible that the context of the miR159 binding site in *MYB33*/*MYB65* denotes them as sensitive targets to miR159 regulation, and thereby is a strong contributing factor to this narrow miR159 functional specificity. This could have broad implications for miRNA target predictions and artificial miRNA design, both of which are solely based on sequence complementarity, but in which contextual features appear to have a strong impact on the silencing outcome [39], [40].

Furthermore, we have recently shown that the efficacy of miR159 in silencing *MYB33* is tissue specific, where it has much weaker efficacy in the seed relative to the rosette [41]. As the miR159-*MYB33* relationship has been conserved for many millions of years, it is conceivable that higher orders of regulation have arisen during this time. Possible factors, such as RNA secondary structures and RNA binding proteins have proven to play a critical role in both specificity and regulation of the silencing outcome in animals [42]–[44]. However, to date these features have been virtually ignored in plants. We believe our findings warrant attention to these possibilities.

### A molecular model for plant miRNA mediated gene silencing

**Figure 12:**
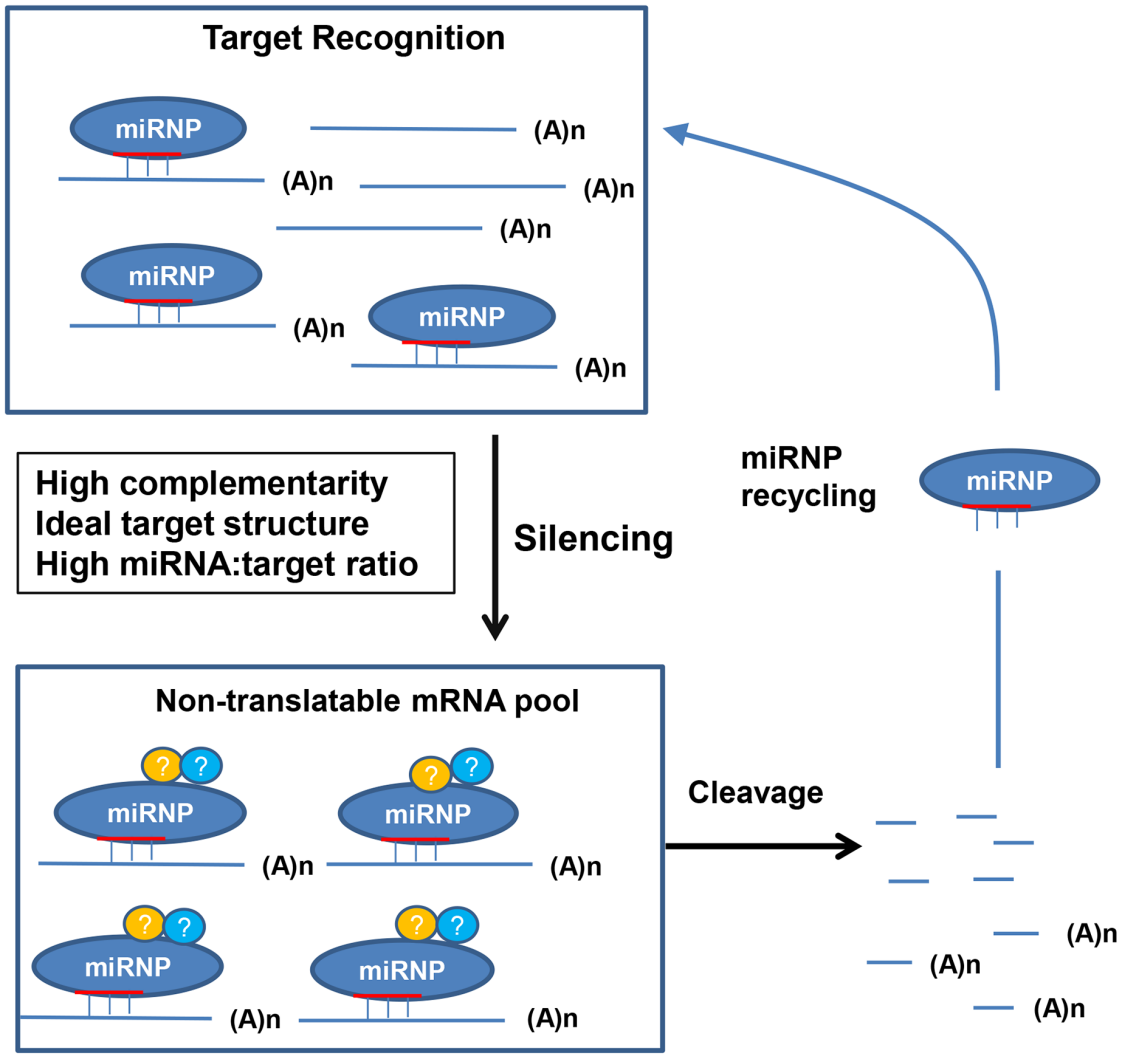
A molecular model for plant miRNA-mediated gene silencing. For *MIR159a1* or *MIR159a2* plants, introduction of central mismatches will increases miRISC-mRNA complex half-life due to an attenuated cleavage efficiency, and so leading to an increased target transcript abundance (Figure 1–4). However, as miR159-guided cleavage fails to clear *MYB33/MYB65* transcripts (Figure 2), especially when *MYB33* is highly transcribed (Figure 5), a non-cleavage (possibly translational repression) mechanism may be the initial or default state of miR159-mediated silencing, with cleavage being a secondary but inherently linked mechanism completing the silencing process, and simultaneously enabling miRISC recycling. When miRISC recycling does become limiting, the miRNA:target mRNA stoichiometry become an increasingly important factor. For instance, attenuated miR159 cleavage of the highly abundant *MYB33-1 cm* and *MYB33-2 cm* transcripts (Figure 7), may result in the miR159-RISC complex becoming limiting, leading to the inefficient silencing of these transgenes (Figure 7, 11). Finally, the non-ideal target structure of the *MYB33-FM* transgene, results in its poor recognition by miR159, enabling this transgene to be expressed (Figure 10, 11).

A molecular model for plant miRNA-mediated target recognition and subsequent silencing is proposed to explain our results (Figure 12). The nascent target transcripts are recognized and bound by the miRNA-loaded RISC (miRISCs). The ability/efficiency of the miRISCs to recognize target mRNA is influenced by at least three factors: the miRNA binding site complementarity, the target mRNA structure/accessibility (which together make the miRNA target site), and the relative concentration of target mRNA to the miRNA, which becomes increasingly important when cleavage, or recognition is attenuated. Inefficient target recognition can be caused by negative changes to any of the above factors solely, or combinatorially, and lead to a prolonged duration before the target gets bound by the miRISCs enabling translation. If the target mRNA gets recognized and bound by the miRISC, it becomes immediately non-translatable and hence silenced. Cleavage occurs from this pool of miRISC-mRNA complexes, the half-life of which is determined by the cleavage efficiency, which is strongly affected by the complementarity of the miRNA–target duplex, especially at positions 10 and 11. As it has been shown that miRISCs can direct multiple rounds of mRNA cleavage *in vitro* [45], [46], a major outcome of this event is not only to irreversibly destroy the target transcript but to recycle the miRISC so that it may participate in further target silencing. Of course additional experiments will be needed to determine how accurate this model is.

## Materials and Methods

### Plant materials and growth conditions

*Arabidopsis thaliana* ecotype Columbia-0 (Col-0) was used in all experiments and is referred to as wild type. The *myb33* mutant used is in ecotype Col-6 with a *glaborous1* background mutation which has no trichomes [30] and the *mir159ab* mutant is as previously described [20]. Plants were grown on soil (Debco Plugger soil mixed with Osmocote Extra Mini fertilizer at 3.5 g/L) either under long day conditions (16 hr light/8 hr dark, 150 µmol/m^2^/sec at 22°C), or under short-day conditions (12 hr light/12 hr dark at 150 µmol/m^2^/sec at 22°C).

### The generation of binary vectors and transgenic plants

For complementation of *mir159ab*, a 3642 bp genomic *MIR159a* (AT1G73687) fragment including the miR159 stem loop region and its extensive 5′ and 3′ flanking sequences, was PCR amplified from *Arabidopsis* genomic DNA with primers containing *attB* sites, and sub-cloned into the Gateway donor vector pDONOR/ZEO (Invitrogen) by performing BP reaction using BP Clonase II enzyme mix (Invitrogen). A mutagenesis approach based on Liu and Naismith [47] was then used to generate entry vectors for miR159a0, miR159a1, miR159a2, miR159a3 and miR159a4 variants. Two pairs of primers were designed for each variant to mutate the corresponding miR159a* and miR159a sequences, by performing PCR on 100 ng of *MIR159a* entry vector, following the setting of 1 cycle of 98°C for 2 min, 20 cycles of 98°C/10 sec, 55°C/30 sec and 72°C for 30 sec/kb extension time, finished with 1 cycle of 55°C/5 min, 72°C/10 min. Each pair of forward and reverse primers contained non-overlapping sequence at 3′ end and primer-primer complementary sequences at the 5′ end, to minimize primer dimerization and enable primers to use the PCR product as template. The non-overlapping sequences were larger than the complementary sequence and had a 5–10°C higher melting temperature. The mutations were placed in both the complementary region and non-overlapping region. The subsequent PCR product was digested with 2 µl *DpnI* enzyme at 37°C for five hours and purified using Wizard SV Gel and PCR Clean-Up System (Promega), and transformed into *E. coli* Alpha-Select Gold Efficiency competent cells (Bioline). All entry vectors were confirmed by diagnostic restriction enzyme digestion and sequencing, and were subjected to LR reaction with pMDC100 vector [48] to generate the corresponding binary vectors.

For *MYB33* variants, a 4356 bp genomic fragment of *MYB33* (AT5G06100), containing identical genomic elements to *MYB33:GUS* [30], was amplified by PCR from genomic DNA and sub-cloned into pDONOR/ZEO (Invitrogen) by BP reaction. This contained 1991 bp of genomic sequence upstream of the *MYB33* start codon, the whole *MYB33* coding region, and 585 bp of sequences 3′ of the *MYB33* stop codon. The subsequent *MYB33* entry vector was mutated using the same strategy described above, to generate the *MYB33-0 cm*, *MYB33-1 cm*, *MYB33-2 cm*, and the *MYB33-FM* entry vectors respectively. For the *mMYB33* construct, the mutagenesis was performed on a *MYB33* entry vector with a 24 bp Strep-II tag coding sequence inserted in front of the stop codon. After confirmation by diagnostic restriction enzyme digestion and sequencing, all these entry vectors were subjected to LR reaction with pMDC123 vector [48] to generate the corresponding binary vectors.

The *MYB33:GUS* and *mMYB33:GUS* constructs were generated previously [30]. For the generation of *MYB33-1 cm:GUS*, *MYB33-2 cm:GUS* and *MYB33-FM:GUS* translational fusions, the GUS gene was cleaved out of the *MYB33:GUS* construct with *NcoI* and ligated into the *NcoI* site of *MYB33-1 cm*, *MYB33-2 cm* and *MYB33-FM*, respectively. This resulted in the GUS gene being fused in frame to the coding region of *MYB33-1 cm*, *MYB33-2 cm* and *MYB33-FM*, respectively, 55 amino acids from the end of the gene. A clone containing the *GUS* gene in the right orientation and with the correct sequence was identified for each construct.

All expression vectors were transformed into *Agrobacterium tumefaciens* strain GV3101 by electroporation [49]. Using the floral dip method [50], *MIR159a* and its five variant constructs were transformed into the *mir159ab* mutant; while all *MYB33*-related constructs were transformed into *myb33*.

Phusion High Fidelity DNA Polymerase (Finnzymes) was used in all PCR reactions following the standard protocol provided by the manufacturer unless stated elsewhere. All primers used were listed in supplementary file, Table S2.

### Expression analysis

TRIzol (Invitrogen) was used for RNA extraction of tissues from plants at different growth stages. The extraction procedure was carried out as per manufacturer's instructions except the following modifications: (1) Approximately 500 mg of plant material was used with 1 mL of Trizol reagent for each extraction; (2) Homogenization of tissues was carried out using a mortar and pestle; (3) The chloroform extraction step was repeated once; (4) Precipitation of RNA was carried out overnight at −20°C to maximize the recovery of small RNAs. RQ1 RNase-Free DNase (Promega) was used to treat RNA samples for qRT-PCR, except those for Taqman sRNA assays. 30–50 µg of total RNA was treated for each sample in a 100 µL reaction volume following the protocol provided, with the addition of RNaseOutRecobinant RNase Inhibitor (Invitrogen) at a concentration of 1 µL/10 µg RNA. Treated RNA was then purified using Spectrum Plant Total RNA Kit (Sigma Aldrich) following instructions from the manufacturer's manual. cDNA synthesis was carried out using SuperScript III Reverse Transcriptase (Invitrogen) and an oligo dT primer according to manufacturer's protocol. For each sample, 250 ng - 5 µg of total RNA was used. The 20 µL reaction was then diluted 50 times in nuclease free distilled water and used for subsequent qRT-PCR. For qRT-PCR, Platinum Taq DNA Polymerase (Invitrogen) with SYB Green (Sigma) and dNTPs (Fisher Biotec) added was used as a master mix. 10 µL of each cDNA sample was added to 9.6 µL of SYB/Taq master mix with 0.4 µL of forward and reverse primers at 10 µmol each. All qRT-PCR reactions (for both reference and genes of interests) were carried out on a Rotor-Gene Q real time PCR machine (QIAGEN) in triplicate, under the following cycling conditions: 1 cycle of 95°C/5 min, 45 cycles of 95°C/15 sec, 60°C/15 sec, 72°C/20 sec. Fluorescence was acquired at the 72°C step. A 55°C to 99°C melting cycle was then carried out. *CYCLOPHILIN 5* (At2g29960) was used to normalise mRNA levels using the comparative quantitation program in the Rotor-Gene Q software package provided by QIAGEN. The value for each gene represents the average of triplicate assays.

### QRT-PCR assays for mature miRNAs

Customized Taqman sRNA assays (Applied Biosystem) were used to quantitate the mature miR159a variants following protocols described by Allen et al., (2010). Each cDNA sample was assayed in triplicate using a Rotor-Gene Q real time PCR machine (QIAGEN) under the following cycling conditions: 1 cycle of 95°C/5 min, 45 cycles of 95°C/15 sec, 60°C/15 sec, 72°C/20 sec. Fluorescence was acquired at the 72°C step. Expression of all miR159a variants were normalized with *sno101* using the comparative concentration analysis program from Rotor-Gene Q software (QIAGEN). The specificity of the assay was tested by performing miR159a2 assays on RNA from *mir159ab*, *MIR159a0* and *MIR159a1* plants, and only background signals were detected (data not shown).

### GUS staining

*In situ* GUS staining was performed on 8-day-old seedlings using the method previously described [30] with the following modifications: seedlings were collected and fixed with 90% acetone for 20 minutes at room temperature, followed by a wash with GUS staining buffer containing 50 mM Na phosphate buffer, pH 7.2, 0.2% Triton X-100, 2 mM potassium ferricyanide and 2 mM potassium ferrocyanide. Histochemical reactions were performed with 2 mM X-Gluc (5-bromo-4-chloro-3-indolyl-β-D-glucuronide) in GUS staining buffer at 37°C overnight. All fixative and substrate solutions were introduced into the plants with a 10–15 min vacuum infiltration. Plants were cleared with 70% ethanol for easy GUS observation.

### MUG assays

MUG assays were performed using 12-day-old seedlings. 50–100 seedlings were ground into a fine powder in liquid N_2_ using a mortar and pestle and homogenized with 450 µl of GUS extraction buffer (0.5 M NAPO4, 0.5 M EDTA, 10% SDS, 10% Triton X-100, 1 M DTT) followed by centrifugation at 4°C for 10 min at 12,000 rpm. Subsequent supernatant containing total protein was transferred into a new microfuge tube and the protein concentration was determined by a standard Bradford assay. A GUS fluorometric assay was prepared in a microtitre plate using 20 µg of protein, 90 µl of GUS Assay buffer (1 mM 4-methylumbelliferyl-*β*-d-glucuronic acid (MUG) in GUS Extraction buffer) and GUS Extraction buffer to a final volume of 150 µl. GUS activity was determined by measuring fluorescence of 4-methylumbelliferone (4-MU) using a Fluostar Fluorometer. Successive fluorescence readings were determined at a wavelength of 355/460 nm over a two hours interval. GUS activity was determined from a set of 4-MU standards and expressed in nmols 4-MU/min/mg protein.

### Modified 5′-Rapid Amplification of cDNA Ends (5′-RACE) of degraded *MYB33* transcripts

RNA was extracted from either inflorescences or rosettes, and treated with DNaseI and purified as described above. A GeneRacer Kit (Invitrogen) was used to ligate 5 µg of the total purified RNA directly to 1 µg of the RNA oligo adapter provided, without carrying out the de-capping procedure described in the manual. After a one hour incubation at 37°C, the ligation mixture was diluted with 90 µL nuclease free distilled water, and 100 µL of phenol∶chloroform was added and was then vortexed vigorously. The aqueous phase was recovered by centrifugation at 14,680 rpm at room temperature for 5 min, and precipitated with 2 µL 10 mg/mL mussel glycogen, 10 µl 3 M sodium acetate pH 5.2 and 220 µL 95% ethanol overnight at −20°C. The RNA was pelleted by centrifugation at 14,680 rpm for 20 min at 4°C. After one wash with 70% ethanol, the pellet was dried and resuspended in 11 µL water. 1 µL of ligated RNA was analysed on a 1% agarose gel by electrophoresis. The remaining 10 µL was retro-transcribed in a 20 µL reaction. The cDNA synthesized (20 µL) was diluted 25 times, and 25 µL of this diluted cDNA was used in a 50 µL PCR reaction with nested GeneRacer oligo-specific and *MYB33*-specific primer. PCR was carried out using Platinum Taq DNA Polymerase (Invitrogen) using the setting of 1 cycle of 94°C/2 min; 30 cycles of 95°C/30 sec, 60°C/30 sec, 72°C/1–2 min; 1 cycle of 72°C for 5 min. The PCR products obtained were purified using Wizard SV Gel and PCR Clean-Up System (Promega) and sequenced using a *MYB33* specific primer downstream the miR159 cleavage site.

## Supporting Information

Figure S1 The two mismatches at positions 7 and 20 between A*rabidopsis* miR159 and *MYB33/MYB65* are conserved across monocots and dicots. Red: mismatches. At: *Arabidopsis thaliana*, Tm: *Triticum monococcum*; Hv: *Hordeum vulgare*; Tt: *Triticum turqidum*; Ta: *Triticum aestivum*; As: *Avena sativa*; Os: *Oryza sativa*; Sb: *Sorghum biocolor*; Vv: *Vitis vulpine*; Zm: *Zea mays*; Pt: *Populus trichocarpa*; Rc: *Ricinus communis*.
(TIF)

Figure S2 Sequence changes made on miR159a* for all miR159a variant constructs. All miR159a*s (a0*–a4*) aligned with corresponding miR159a variant (a0–a4) in the orange box. Red: mutated nucleotides.
(TIF)

Figure S3 Modified 5′ end RACE of miR159-guided 3′ *MYB33* cleavage product. (A) Schematic representation of a modified 5′-RACE procedure to determine the proportion of degraded 3′ *MYB33* mRNA that corresponds to miR159-guided cleavage products. (B) miR319-cleaved *MYB33* transcripts were co-recovered with the miR159-cleavege products in *mir159ab*. The cleavage site of miR319 is one bp upstream of the miR159 cleavage site, and sequencing corresponding to miR319-guided cleavage products is apparent due to the one nucleotide shift in the location of the adapter sequence as can be read in the trace file. Nucleotides in blue are from miR319-cleavage products, while black ones are from miR159 cleavage products.
(TIF)

Figure S4 Increased number of mismatches is correlated with higher target transcript levels in *mir159ab* plants complemented by the miR159a variants. (A) The average *MYB33* mRNA levels in *MIR159a0*, *a1* and *a2* plants. (B) The average *MYB65* mRNA levels in *MIR159a0*, *MIR159a1* and *MIR159a2* plants. All mRNA levels were normalized with *CYCLOPHILIN 5*. Measurements are the average of three transgenic lines. Error bars represent the SEM.
(TIF)

Figure S5 Specificity test on the qRT-PCR assay developed to quantitate miR159-guided *MYB33* 3′ cleavage products. (A) Design of the four forward primers used in qRT-PCR to quantitate miR159-guided *MYB33* 3′-end cleavage product levels. All the nucleotides corresponding to the adaptor sequence are indicated as red. *MYB33* sequences downstream of miR159 cleavage site are highlighted as blue, while upstream sequences are green. (B) Gel electrophoresis of products from the control assays to test the specificity of the *MYB33* cleavage assay in Col-0 and *mir159ab*. Products were amplified by qRT-PCR using three different *MYB33*-specific forward primers, CS-F, CS-1F and CS+1F respectively with an identical reverse primer. (C) Results of control assays in Col-0 and *mir159ab* to test the primer specificities. All mRNA levels were normalized with *CYCLOPHILIN* with measurements being the average of three replicates. Error bars represent the SEM. An independent biological replication had the same trend. No product can be amplified using CS+2F as the forward primer.
(TIF)

Figure S6 A more extensive alignment of nucleotide sequences of *MYB33/MYB65* homologues showing conservation of nucleotide sequences. Nucleotides encoding the R2R3 MYB domain are boxed in red and the miR159 binding domain is boxed in blue.
(DOC)

Table S1 Hybridization free energy comparison for different miR159 and *MYB33* variant pairs.
(XLSX)

Table S2 Primer table.
(XLSX)
