# Supplementary figures and images for: Determinants beyond Both Complementarity and Cleavage Govern MicroR159 Efficacy in Arabidopsis
Source: PLoS Genet. 2014 Mar 13;10(3):e1004232. doi: 10.1371/journal.pgen.1004232 (PMC3953016; doi:10.1371/journal.pgen.1004232)

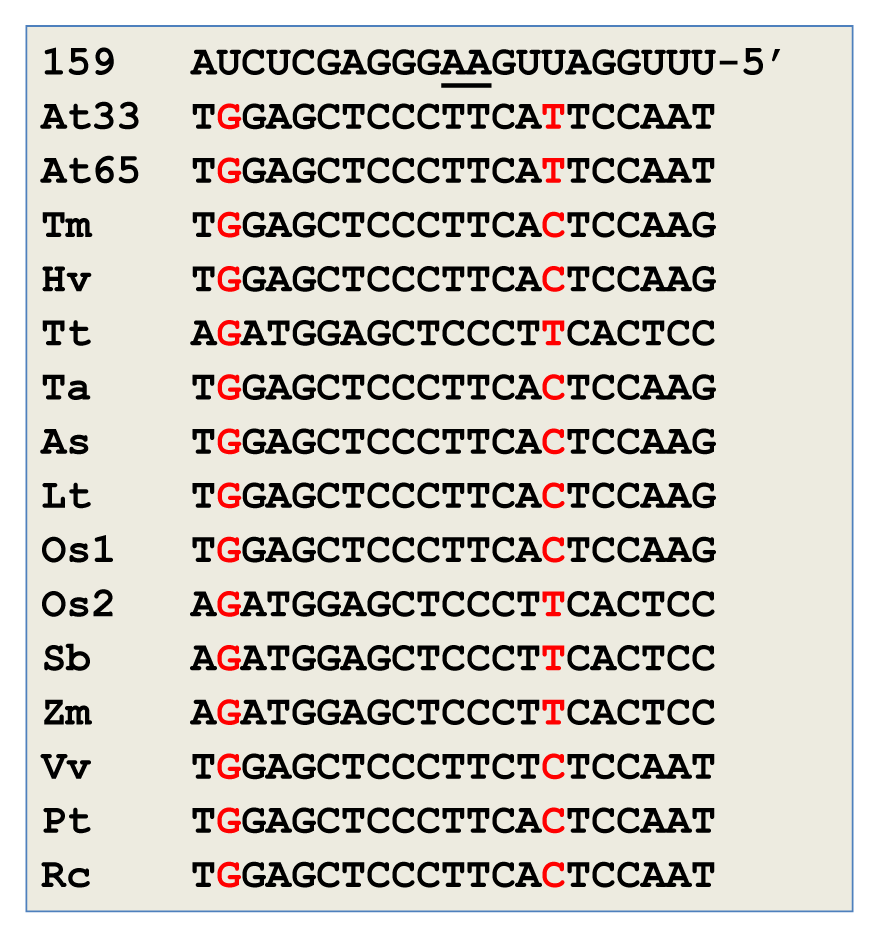

Supplement: Figure S1 — The two mismatches at positions 7 and 20 between Arabidopsis miR159 and MYB33/MYB65 are conserved across monocots and dicots. Red: mismatches. At: Arabidopsis thaliana, Tm: Triticum monococcum; Hv: Hordeum vulgare; Tt: Triticum turqidum; Ta: Triticum aestivum; As: Avena sativa; Os: Oryza sativa; Sb: Sorghum biocolor; Vv: Vitis vulpine; Zm: Zea mays; Pt: Populus trichocarpa; Rc: Ricinus communis. (TIF) [file pgen.1004232.s001.tif]

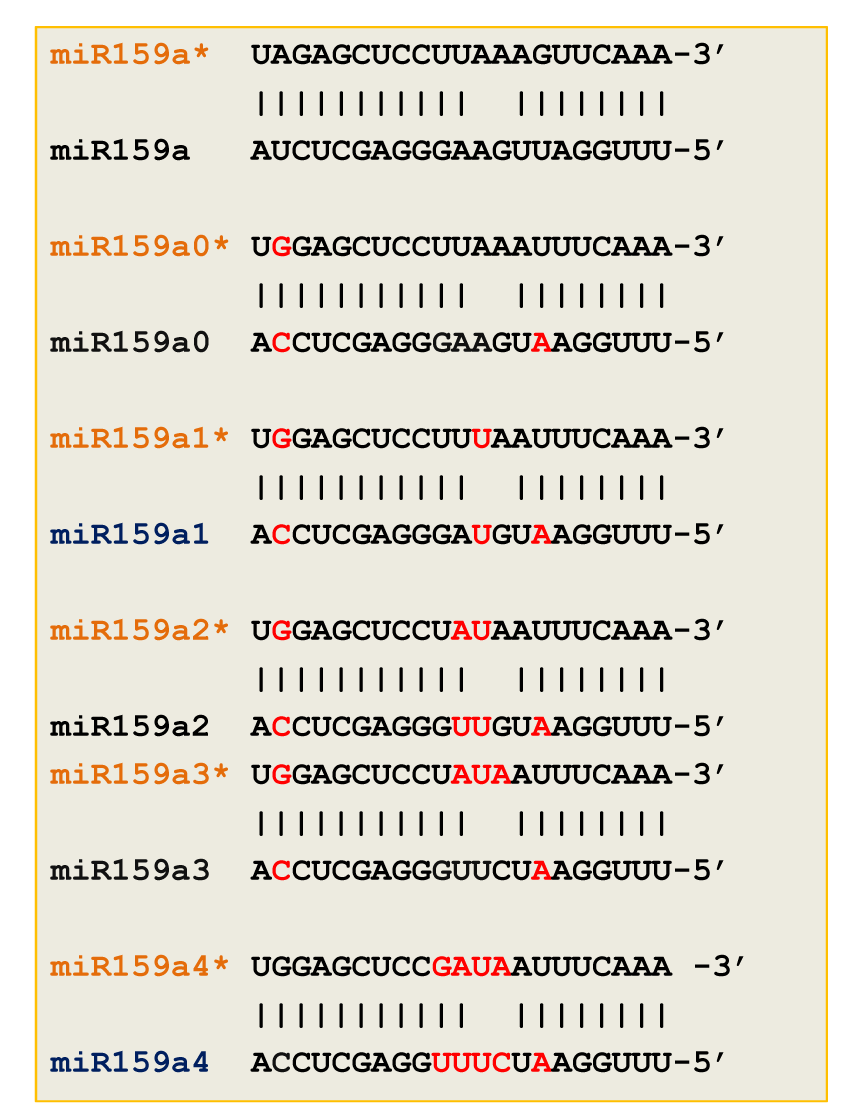

Supplement: Figure S2 — Sequence changes made on miR159a* for all miR159a variant constructs. All miR159a*s (a0*–a4*) aligned with corresponding miR159a variant (a0–a4) in the orange box. Red: mutated nucleotides. (TIF) [file pgen.1004232.s002.tif]

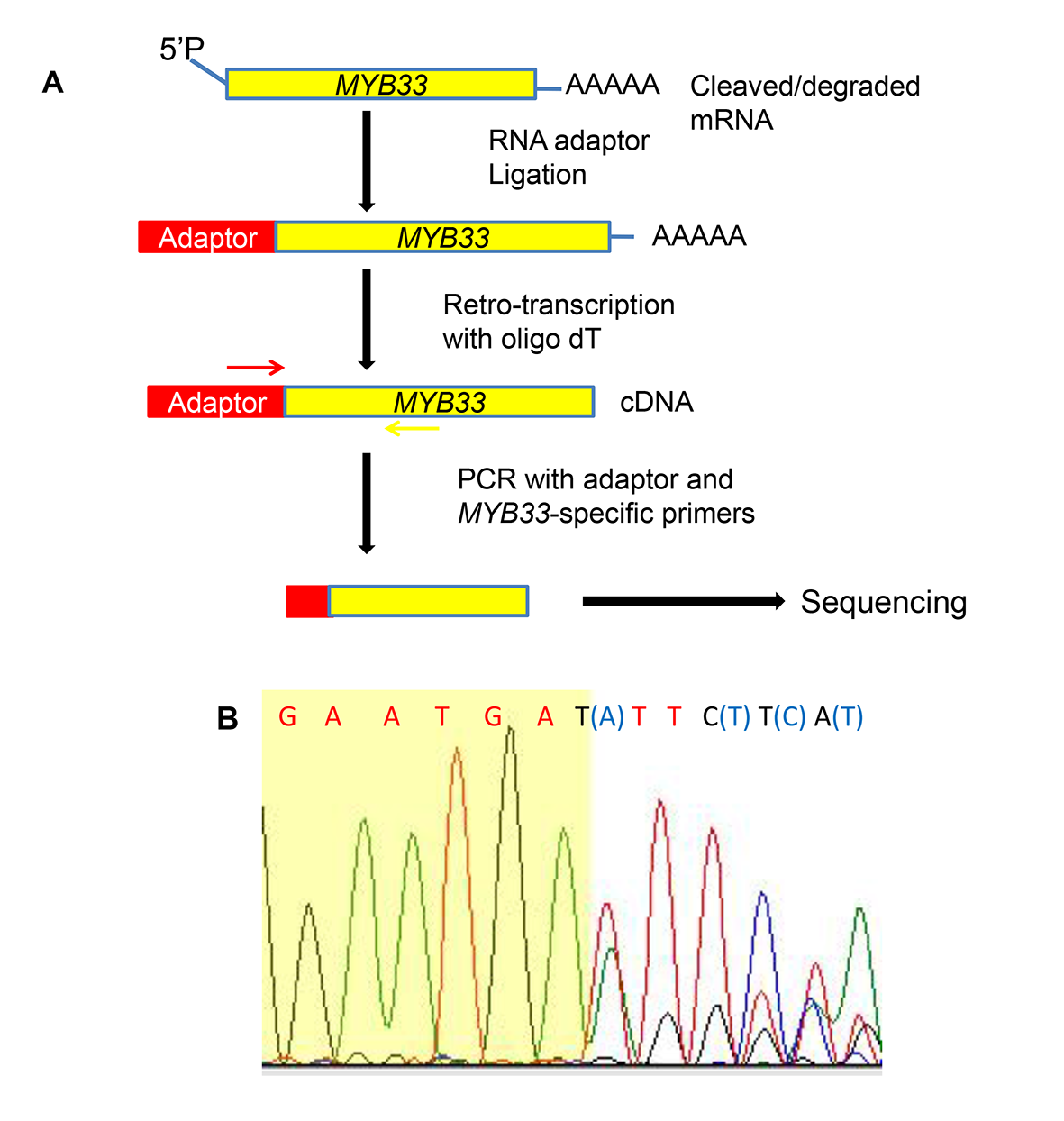

Supplement: Figure S3 — Modified 5′ end RACE of miR159-guided 3′ MYB33 cleavage product. (A) Schematic representation of a modified 5′-RACE procedure to determine the proportion of degraded 3′ MYB33 mRNA that corresponds to miR159-guided cleavage products. (B) miR319-cleaved MYB33 transcripts were co-recovered with the miR159-cleavege products in mir159ab. The cleavage site of miR319 is one bp upstream of the miR159 cleavage site, and sequencing corresponding to miR319-guided cleavage products is apparent due to the one nucleotide shift in the location of the adapter sequence as can be read in the trace file. Nucleotides in blue are from miR319-cleavage products, while black ones are from miR159 cleavage products. (TIF) [file pgen.1004232.s003.tif]

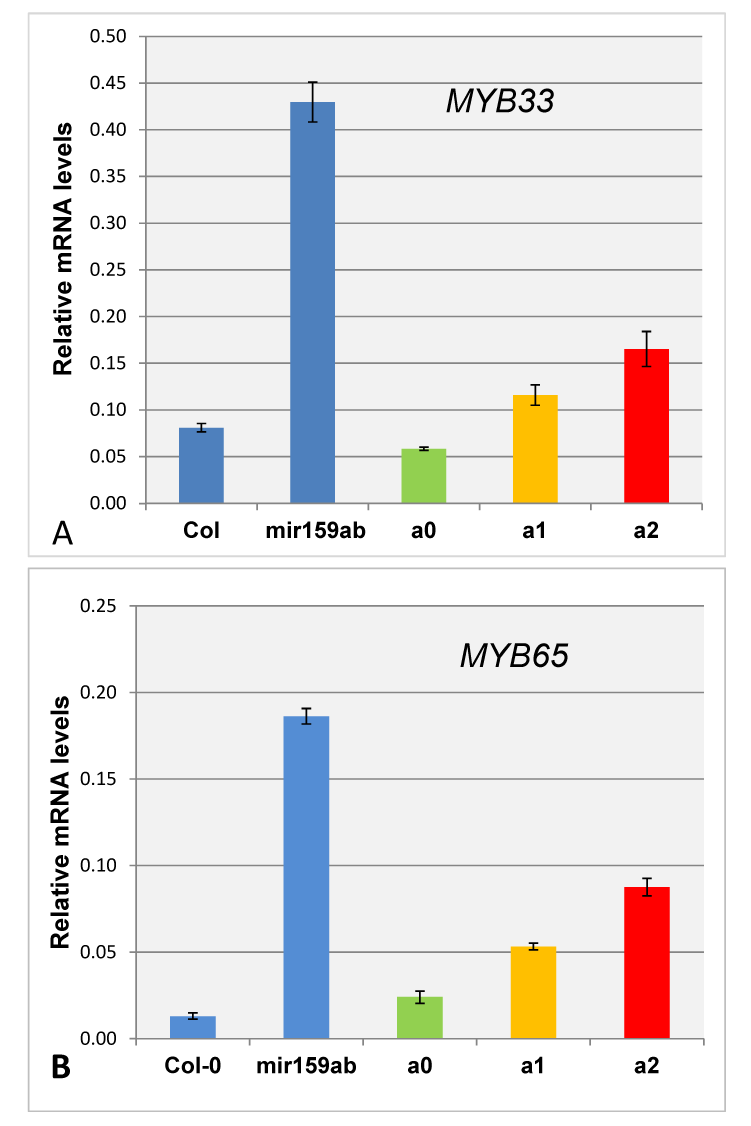

Supplement: Figure S4 — Increased number of mismatches is correlated with higher target transcript levels in mir159ab plants complemented by the miR159a variants. (A) The average MYB33 mRNA levels in MIR159a0, a1 and a2 plants. (B) The average MYB65 mRNA levels in MIR159a0, MIR159a1 and MIR159a2 plants. All mRNA levels were normalized with CYCLOPHILIN 5. Measurements are the average of three transgenic lines. Error bars represent the SEM. (TIF) [file pgen.1004232.s004.tif]

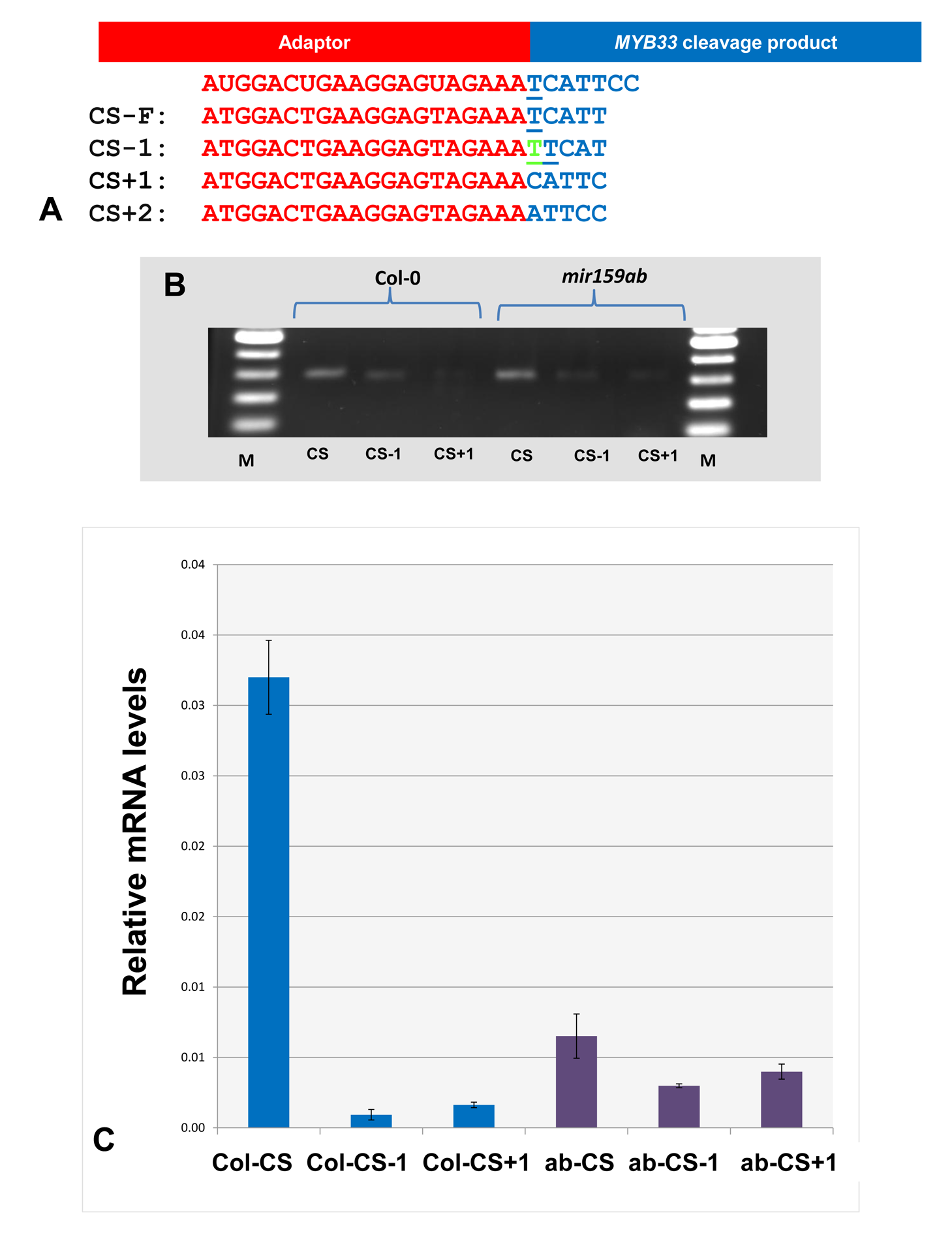

Supplement: Figure S5 — Specificity test on the qRT-PCR assay developed to quantitate miR159-guided MYB33 3′ cleavage products. (A) Design of the four forward primers used in qRT-PCR to quantitate miR159-guided MYB33 3′-end cleavage product levels. All the nucleotides corresponding to the adaptor sequence are indicated as red. MYB33 sequences downstream of miR159 cleavage site are highlighted as blue, while upstream sequences are green. (B) Gel electrophoresis of products from the control assays to test the specificity of the MYB33 cleavage assay in Col-0 and mir159ab. Products were amplified by qRT-PCR using three different MYB33-specific forward primers, CS-F, CS-1F and CS+1F respectively with an identical reverse primer. (C) Results of control assays in Col-0 and mir159ab to test the primer specificities. All mRNA levels were normalized with CYCLOPHILIN with measurements being the average of three replicates. Error bars represent the SEM. An independent biological replication had the same trend. No product can be amplified using CS+2F as the forward primer. (TIF) [file pgen.1004232.s005.tif]
