# Supplementary material for: Determinants beyond Both Complementarity and Cleavage Govern MicroR159 Efficacy in Arabidopsis
Source: PLoS Genet. 2014 Mar 13;10(3):e1004232. doi: 10.1371/journal.pgen.1004232 (PMC3953016; doi:10.1371/journal.pgen.1004232)
Supplement: Figure S6 — A more extensive alignment of nucleotide sequences of MYB33/MYB65 homologues showing conservation of nucleotide sequences. Nucleotides encoding the R2R3 MYB domain are boxed in red and the miR159 binding domain is boxed in blue. (DOC) [file pgen.1004232.s006.doc]

**Sequence alignment MYB33/65 homologues**

Sb -----------ATGTACCGGGTGAAAAGCGAGGG------------CGAGGGCGACTGCG 37

Zm GCCAGCGCGCGATGTACCGGGTGAAGAGCGAGGGGGAGGGCGAGGGCGAGGGCGACTGCG 340

Hv -----------ATGTACCGGGTGAAGAGCGAGAG------------------CGACTGCG 31

Tt ACGGAGAG---ATGTACCGGGTGAAGAGCGAGAG------------------CGACTGCG 816

Ta ACGGAGAG---ATGTACCGGGTGAAGAGCGAGAG------------------CGACTGCG 49

Tm ACGGAGAG---ATGTACCGGGTGAAGAGCGAGAG------------------CGACTGCG 185

Os1 GTTGAGACGCCATGTATCGGGTGAAGAGCGAGAG------------------CGACTGCG 335

Os2 GTTGAGACGCCATGTATCGGGTGAAGAGCGAGAG------------------CGACTGCG 426

Al AATTGTTGCTCAAGTACTGCTTTAATTAGAAAGG---------------AGATGAGTTAC 238

Lt AATTGTTGCTCAAGTACTGCTTTAATTAGAAAGG---------------AGATGAGTTAC 238

AtMYB33 ---------------------------------------------------ATGAGTTAC 9

AtMYB65 --------------------------------------------------GATGA-TGGT 39

Rc ATTACAAGGAGATGAGTCACACGACAAATGAGAG---------------TGATGA-TGGG 413

Pt -----------ATGAGTCGCACGACAAGTGAGAG---------------TGAGGA-TGGC 33

** *

Sb AGATGATGCTGCAGGAC---CAGATGGACTCGCCGGTGGCCGACGACGTGAGCAGCGGAG 94

Zm AAATGATGCTGCAGGAA---CAGATGGACTCGCTGGTGGCCGACGACGTCAGCAGCGGAG 397

Hv AGATGATGCACCAGGAGGACCAGATGGACTCGCCGGTGGGCGACGACGGCAGCAGCGGCG 91

Tt AGATGATGCATCAGGAGGACCAGATGGACTCGCCGGTGGGCGACGACGGCAGCAGCG--- 873

Ta AGATGATGCATCAGGAGGACCAGATGGACTCGCCGGTGGGCGACGACGGCAGCAGCG--- 106

Tm AGATGATGCACCAGGAGGACCAGATGGACTCGCCGGTGGGCGACGACGGCAGCAGCG--- 242

Os1 ATATGATCCATCAGGAG---CAGATGGACTCGCCGGTGGCCGACGACGGCAGCAGCG--- 389

Os2 AGATGATCCATCAGGAG---CAGATGGACTCGCCGGTGGCCGACGACGGCAGCAGCG--- 480

Al ACGAGCACTGACAGTGA--CCATAATGAGTCACCAGTTGCTGATGATAATGGAAGTGACT 296

Lt ACGAGCACTGACAGTGA--CCATAATGAGTCACCAGTTGCTGATGATAATGGAAGTGACT 296

AtMYB33 ACGAGCACTGACAGTGA--CCATAATGAGTCACCAGCTGCTGATGATAATGGAAGTGACT 67

AtMYB65 AT--GCACTC-CAGCAT--CCATAATGAATCACCAGCTCCTGATAGTATTAGCAATGGCT 94

Rc CT--GTTCTCTAAG-GA--TCGGATTGATTCACCATTGGCTGAGGGA---GGTAACTGCG 465

Pt AT--GATCTCCAAG-GA--TCAGACTGGGTTGCCATTGGGCGAGGAA---GGCAGCTATG 85

* ** * * * * * ** * *

Sb GCGGGTCGCCTCATCCTCACAGGGGCGCCGGGCCGCCCCTGAAGAAAGGGCCATGGACGT 154

Zm GAGGGTCGCC------TCACAGGGGCGTCGGCACGCCCCTGAAGAAGGGGCCATGGACGT 451

Hv GAGGGTCGCC------TCACAGGGGCGGCGGGCCGCCTCTGAAGAAGGGGCCCTGGACGT 145

Tt GAGGGTCGCC------TCACAGGGGCGGCGGGCCGCCTCTGAAGAAAGGGCCCTGGACGT 927

Ta GAGGGTCGCC------CCACAGGGGCGGCGGGCCGCCTCTGAAGAAGGGCCCCTGGACGT 160

Tm GAGGGTCGCC------CCACAGGGGCGGCGGGCCGCCTCTGAAGAAGGGCCCCTGGACGT 296

Os1 GGGGGTCGCC------GCACCGCGGCGGCGGGCCCCCGCTGAAGAAGGGGCCATGGACGT 443

Os2 GGGGGTCGCC------GCACCGCGGCGGCGGGCCCCCGCTGAAGAAGGGGCCATGGACGT 534

Al GCAGAA------------GTAGATGGGAAGGTCATGCTCTGAAGAAAGGGCCATGGAGTT 344

Lt GCAGAA------------GTAGATGGGAAGGTCATGCTCTGAAGAAAGGGCCATGGAGTT 344

AtMYB33 GCAGAA------------GTAGATGGGATGGTCATGCTCTCAAGAAAGGCCCTTGGAGTT 115

AtMYB65 GCAGAA------------GTAGAGGGAAAAGAAGTGTCCTGAAGAAAGGACCATGGACTT 142

Rc GAGGAA------------GCGCAAATGGAGGAGCTATGCTGAAGAAAGGGCCATGGACAT 513

Pt GTGGAA------------GTACAAATGGAG---TTGTTCTTAAGAAAGGGCCATGGACTT 130

* * ** ***** ** ** **** *

Sb CCGCGGAGGACGCCATCCTGGTGGACTACGTCAAGAAGCACGGCGAAGGGAACTGGAACG 214

Zm CCGCGGAGGACGCCATCCTGGTGGACTACGTTAAGAAGAACGGCGAGGGCAACTGGAACG 511

Hv CCGCGGAGGACGCCATCCTGGTGGACTACGTGAAGAAGCACGGCGAGGGGAACTGGAACG 205

Tt CGGCGGAGGACGCCATCCTGGTGGACTACGTGAAGAAGCACGGCGAGGGGAACTGGAACG 987

Ta CGGCGGAGGACGCCATCCTGGTGGACTACGTGAAGAAGCACGGCGAGGGGAACTGGAACG 220

Tm CGGCGGAGGACGCCATCCTGGTGGACTACGTGAAGAAGCACGGCGAGGGGAACTGGAACG 356

Os1 CGGCGGAGGACGCCATCCTGGTGGACTACGTGAAGAAGCACGGCGAGGGGAACTGGAACG 503

Os2 CGGCGGAGGACGCCATCCTGGTGGACTACGTGAAGAAGCACGGCGAGGGGAACTGGAACG 594

Al CTGCTGAAGATGATATTCTTATTGACTATGTGAATAAGCATGGTGAAGGTAACTGGAATG 404

Lt CTGCTGAAGATGATATTCTTATTGACTATGTGAATAAGCATGGTGAAGGTAACTGGAATG 404

AtMYB33 CAGCTGAAGATGATATTCTTATTGACTATGTGAATAAGCATGGTGAGGGTAACTGGAATG 175

AtMYB65 CAACTGAAGACGGGATTTTAATTGATTATGTAAAGAAGCACGGCGAGGGTAACTGGAATG 202

Rc CTGCTGAAGATGCAATTTTGATAGAATATGTGAAGAAACATGGGGAGGGGAACTGGAATG 573

Pt CTGCTGAAGATGCAATTTTGATAGAATATGTGAAGAAGCACGGGGAGGGGAATTGGAATT 190

* * ** ** * ** * * ** ** ** ** ** * ** ** ** ** *****

Sb CGGTGCAGAAGAACACCGGGCTGTTCCGCTGCGGCAAGAGCTGCCGCCTCCGCTGGGCGA 274

Zm CGGTGCAGAAGAACACCGGGCTGTTCCGCTGCGGCAAGAGCTGCCGCCTCCGGTGGGCGA 571

Hv CGGTGCAGAAGAACACCGGGCTGTTCCGGTGCGGCAAGAGCTGCCGCCTCCGGTGGGCGA 265

Tt CGGTGCAGAAGAACACCGGGCTGTTCCGCTGCGGTAAGAGCTGCCGCCTCCGGTGGGCGA 1047

Ta CGGTGCAGAAGAACACCGGGTTGTTCCGGTGCGGCAAGAGCTGCCGCCTCCGGTGGGCGA 280

Tm CGGTGCAGAAGAACACCGGGCTGTTCCGGTGCGGCAAGAGCTGCCGCCTCCGGTGGGCGA 416

Os1 CGGTGCAGAAGAACACCGGGCTGTTCCGGTGCGGCAAGAGCTGCCGCCTCCGGTGGGCGA 563

Os2 CGGTGCAGAAGAACACCGGGCTGTTCCGGTGCGGCAAGAGCTGCCGCCTCCGGTGGGCGA 654

Al CTGTGCAGAGACATACGGGCTTGTTTCGTTGTGGTAAAAGCTGTCGTCTAAGGTGGGCTA 464

Lt CTGTGCAGAGACATACGGGCTTGTTTCGTTGTGGTAAAAGCTGTCGTCTAAGGTGGGCTA 464

AtMYB33 CTGTGCAGAAACACACCAGCTTGTTTCGTTGTGGTAAAAGCTGTCGTCTAAGATGGGCTA 235

AtMYB65 CTGTGCAGAAACACACTAGCCTGGCCCGTTGTGGTAAAAGCTGTCGTCTGAGATGGGCTA 262

Rc CCGTTCAGAAGCACTCAGGACTTTCTCGCTGTGGCAAAAGCTGCAGATTGCGATGGGCCA 633

Pt CTGTTCAGAAGCACTCAGGGCTTTTCCGTTGCGGTAAAAGCTGCAGATTACGATGGGCCA 250

* ** **** * * * * ** ** ** ** ** ** * * * ***** *

Sb ACCACCTCAGGCCCAACCTCAAGAAGGGGGCCTTCACCCCGGAGGAGGAGCGCCTCATCA 334

Zm ACCACCTCAGGCCCAACCTCAAGAAGGGGGCCTTCACCCCCGAGGAGGAGCGCCTCATCA 631

Hv ACCACCTCAGGCCCAACCTCAAGAAGGGGGCCTTCACCCCCGAGGAGGAGAGGCTCATCA 325

Tt ACCACCTCAGGCCCAACCTCAAGAAGGGGGCCTTCACCCCCGAGGAGGAGAGGCTCATCA 1107

Ta ACCACCTCAGGCCCAACCTCAAGAAGGGGGCCTTCACCCCCGAGGAGGAGAGGCTCATCA 340

Tm ACCACCTCAGGCCCAACCTCAAGAAGGGGGCCTTCACCCCCGAGGAGGAGAGGCTCATCA 476

Os1 ACCACCTGAGGCCCAACCTCAAGAAGGGGGCCTTCACCGCCGAGGAGGAGAGGCTCATCA 623

Os2 ACCACCTGAGGCCCAACCTCAAGAAGGGGGCCTTCACCGCCGAGGAGGAGAGGCTCATCA 714

Al ATCATCTGAGGCCAAATTTGAAGAAAGGAGCGTTTAGTCAAGAAGAAGAACAGCTTATTC 524

Lt ATCATCTGAGGCCAAATTTGAAGAAAGGAGCGTTTAGTCAAGAAGAAGAACAGCTTATTC 524

AtMYB33 ATCATCTGAGGCCAAATTTGAAGAAAGGAGCTTTTAGTCAAGAAGAAGAACAGCTTATCG 295

AtMYB65 ATCATCTGAGGCCAAACTTGAAGAAAGGAGCATTTAGCCAAGAAGAAGAACAGCTCATTG 322

Rc ATCACCTGAGGCCTAATTTGAAAAAAGGGGCATTTACTCAAGAAGAAGAACAGTTAATCA 693

Pt ATCATCTAAGGCCTAATTTAAAGAAAGGAGCATTTACTCATGAAGAAGAGCAACTAATCA 310

* ** ** ** ** ** * ** ** ** ** ** * ** ** ** * **

Sb TCCAGCTCCACGCCAAGATGGGGAACAAGTGGGCAAGGATGGCTGCTCAT---------- 384

Zm TCCAGCTCCACGCCAAGATGGGGAACAAGTGGGCGAGGATGGCTGGTCAC---------- 681

Hv TCCAGCTCCACTCCAAGATGGGCAACAAGTGGGCTCGGATGGCCGCTCAT---------- 375

Tt TCCAGCTCCACTCCAAGATGGGCAACAAGTGGGCTCGGATGGCCGCTCATGTAAGTGCGA 1167

Ta TCCAGCTCCACTCCAAGATGGGCAACAAGTGGGCTCGGATGGCCGCTCAT---------- 390

Tm TCCAGCTCCACTCCAAGATGGGCAACAAGTGGGCTCGGATGGCCGCTCAT---------- 526

Os1 TCCAGCTCCACTCCAAGATGGGGAACAAGTGGGCTCGGATGGCCGCTCAT---------- 673

Os2 TCCAGCTCCACTCCAAGATGGGGAACAAGTGGGCTCGGATGGCCGCTCAT---------- 764

Al TTGAATTGCATGCCAAGATGGGTAATAGATGGGCGCGTATGGCTGCGCAT---------- 574

Lt TTGAATTGCATGCCAAGATGGGTAATAGATGGGCGCGTATGGCTGCGCAT---------- 574

AtMYB33 TTGAATTGCATGCCAAGATGGGTAATAGATGGGCACGTATGGCTGCACAT---------- 345

AtMYB65 TTGAAATGCACGCCAAGATGGGAAATAAATGGGCACAGATGGCTGAACAT---------- 372

Rc TTGAGCTCCATGCCAAGATGGGAAACAAATGGGCACGCATGGCTGCACAT---------- 743

Pt TTGAACTTCATGCCAAGATGGGAAACAAATGGGCACGGATGGCTGCACAT---------- 360

* * * ** * ******** ** * ***** ***** * **

Sb ------------------------------------------TTTCCAGGGAGAATTGAT 402

Zm ------------------------------------------TTGCCAGGGCGTACTGAC 699

Hv ------------------------------------------TTGCCAGGGCGTACTGAT 393

Tt CACCTCTTGTTGTTATTATTGACTTTGAGCACCTCTTCTCAGTTGCCAGGGCGTACTGAT 2007

Ta ------------------------------------------TTGCCAGGGCGTACTGAC 408

Tm ------------------------------------------TTGCCAGGGCGTACTGAC 544

Os1 ------------------------------------------TTGCCAGGGCGCACTGAT 691

Os2 ------------------------------------------TTGCCAGGGCGCACTGAT 782

Al ------------------------------------------TTGCCTGGCCGAACGGAT 592

Lt ------------------------------------------TTGCCTGGCCGAACGGAT 592

AtMYB33 ------------------------------------------TTGCCTGGCCGAACGGAT 363

AtMYB65 ------------------------------------------TTACCTGGTCGAACAGAT 390

Rc ------------------------------------------TTGCCTGGTCGTACAGAC 761

Pt ------------------------------------------TTGCCTGGTCGTACAGAC 378

** ** ** * * **

Sb GTTGAAATTAAAAATTACTGGAACACTCGAATAAAGAGATGTCAACGAGCTAGCCTTCCT 462

Zm AATGAGATCAAGAACTACTGGAACACTCGAATAAAGAGATGTCAACGAGCTAGCCTTCCT 759

Hv AATGAAATAAAGAATTACTGGAACACTCGAATAAAGAGATGTCAGCGAGCCGGTTTGCCA 453

Tt AATGAAATAAAGAATTACTGGAACACTCGAATAAAGAGATGCCAGCGAGCTGGCTTGCCA 2067

Ta GATGAAATAAAGAATTACTGGAACACTCGAATAAAGAGATGTCAGCGAGCTGGCTTGCCA 468

Tm AATGAAATAAAGAATTATTGGAACACTCGAATAAAGAGATGTCAGCGAGCCGGTTTGCCA 604

Os1 AATGAAATAAAGAATTACTGGAATACTCGAATAAAGAGATGCCAGCGAGCTGGCCTACCC 751

Os2 AATGAAATAAAGAATTACTGGAATACTCGAATAAAGAGATGCCAGCGAGCTGGCCTACCC 842

Al AACGAGATAAAGAATTATTGGAACACTCGTATCAAGAGGCGACAACGAGCTGGTTTACCA 652

Lt AACGAGATAAAGAATTATTGGAACACTCGTATCAAGAGGCGACAACGAGCTGGTTTACCA 652

AtMYB33 AATGAGATAAAGAATTATTGGAACACTCGTATCAAGAGGCGACAACGAGCTGGTTTGCCA 423

AtMYB65 AATGAGATAAAGAATTATTGGAACACTCGTATCAAGAGGAGACAACGAGCAGGCTTACCA 450

Rc AATGAGATAAAGAATTACTGGAACACCAGAATTAAGAGGCGCCAACGGGCTGGTTTACCT 821

Pt AATGAGATAAAGAATTACTGGAATACCAGAATTAAGAGGCGTCAACGGGCTGGCTTACCC 438

** ** ** ** ** ***** ** * ** ***** * ** ** ** * * **

Sb ATCTATCCTGCAAGTGTATGCAATCAATCTTCAAATGAAGATCAGCAAGTGTCTG----- 517

Zm ATTTATCCTGCTAGTGTATGCAATCAATCTACAAATGAAGATCAGCAACTGTCTG----- 814

Hv ATATATCCTGCTAGTGTATGCAATCAATCTTCAAATGAAGATCAGCAGGGCTCCA----- 508

Tt ATATATCCTGCTAGTGTATGCAATCAATCTTCAAATGAAGACCA---GGGCTCCA----- 2119

Ta ATATATCCTGCTAGCGTATGCAACCAATCTTCAAATGAAGATCAGCAGGGCTCCA----- 523

Tm ATATATCCTGCTAGCGTATGCAACCAGTCTTCAAATGAAGATCAGCAGGGCTCCA----- 659

Os1 ATCTATCCTACCAGCGTATGCAATCAATCCTCAAATGAAGATCAGCAGTGCTCCA----- 806

Os2 ATCTATCCTACCAGCGTATGCAATCAATCCTCAAATGAAGATCAGCAGTGCTCCA----- 897

Al CTTTATCCTCCTGAAATGCATGTTGAAGCACTAGATTGGAGTCAAGAGTATGCCAAGA-- 710

Lt CTTTATCCTCCTGAAATGCATGTTGAAGCACTAGATTGGAGTCAAGAGTATGCCAAGA-- 710

AtMYB33 CTTTATCCTCCTGAGATGCATGTTGAGGCACTTGAGTGGAGTCAAGAGTATGCCAAGA-- 481

AtMYB65 CTTTACCCTCCTGAAATCTATGTTGATGACCTTCATTGGAGCGAAGAGTATACAAAGA-- 508

Rc CTATATCCTCCAGAAGTCTCTTTCCAAGCACTGCAGGAGAGCCATCAAGGCCTAACCATT 881

Pt CTTTATCCACCTGAAGTCTCTTTGCAAACTTTGCAGGGGAGTCAACAATGCCTGGACATT 498

* ** ** * * * * *

Sb -GTGATTATAATGGTGGCGAGAACATA------TCCAATGATCTTCTATCCGGGAACAGC 570

Zm -GTAATTTTAACGGTGGCGAGAATATA------TCCAATGATCTTCTATCTGGGAACAGC 867

Hv -GCGATTTCAACTGCGGCGAGAATCTT------TCCAGTGACCTCCTGAATGGAAATGGT 561

Tt -GTGATTTCAACTGCGGCGAGAATCTT------TCCAGTGACCTTCTGAATGGAAATGGC 2172

Ta -GCGATTTCAACTGCGGCGAGAATCTT------TCCAGTGACCTTCTGAATGGAAATGGT 576

Tm -GCGATTTCAACTGCGGCGAAAATCTT------TCCAGCGACCTTCTGAATGGAAATGGC 712

Os1 -GTGATTTTGACTGTGGCGAGAATTTG------TCAAACGATCTTCTGAATGCAAATGGT 859

Os2 -GTGATTTTGACTGTGGCGAGAATTTG------TCAAACGATCTTCTGAATGCAAATGGT 950

Al -GTAGAGTTATGGGAGAAGATGGAAGA------CATCAAGATTTCTTGCAGCTGGGGAGT 763

Lt -GTAGAGTTATGGGAGAAGATGGAAGA------CATCAAGATTTCTTGCAGCTGGGGAGT 763

AtMYB33 -GTAGAGTTATGGGAGAAGATAGAAGA------CATCAAGATTTCTTGCAGCTGGGGAGT 534

AtMYB65 -GTAATATCATAAGAGTAGATAGAAGAAGAAGACATCAAGATTTCTTGCAGTTGGGGAAT 567

Rc GGCGGAATCAATACCGGGGATAAGGTT------CACGGTGATCTCTTGCGCAACAATGGC 935

Pt AACGGAATGGACAGTGGGAATAAAGGT------CAGCATGATATCTTGCAGACCCACAAT 552

* * ** * *

Sb CTTTATCTACCAGATTTTACCAGTGACAATTTCATTGCTAATCCAGAGGCTTTATCATAT 630

Zm CTTTATCTGCCAGATTTTACCAGTGACAATTTCATTGCGAACCCAGAGGCTTTATCCTAT 927

Hv CTTTATCTGCCAGATTTTACCTGTGACAATTTCATTGCTAATTCAGAGGCTCTATCTTAT 621

Tt CTTTATCTACCAGATTTTACCTGTGACAATTTCATTGCTAATTCAGAGGCTTTATCTTAT 2232

Ta CTTTATCTGCCAGATTTTACCTGTGACAATTTCATTGCTAATTCAGAGGCTCTATCTTAT 636

Tm CTTTATCTGCCAGATTTTACCTGTGACAATTTCATTGCTAATTCAGAGGCTTTATCTTAT 772

Os1 CTTTACCTACCAGATTTTACCTGTGACAATTTCATTGCTAATTCAGAGGCTTTACCTTAT 919

Os2 CTTTACCTACCAGATTTTACCTGTGACAATTTCATTGCTAATTCAGAGGCTTTACCTTAT 1010

Al TGTGA---ATCTAATTTCTTCTTTGATAGTCTTAACTTTACAGACA---TGGTACCTGGT 817

Lt TGTGA---ATCTAATTTCTTCTTTGATAGTCTTAACTTTACAGACA---TGGTACCTGGT 817

AtMYB33 TGTGA---ATCTAATGTCTTCTTTGATACTCTTAATTTTACCGACA---TGGTACCTGGT 588

AtMYB65 TCTAA---AGATAATGTCTTATTTGACGATCTAAATTTTGCTGCTAGCTTGTTACCTGCC 624

Rc TATGAGATACCTGATGTCATATTTGACAGTTTAAAAGCAAGCCAAGGCATCTCACCATAT 995

Pt TATGGGATACCTGATGTCATGTTTGACAATTTAAAGACCAACCGAAGCATCTTACCTTAT 612

* ** * ** * * * *

Sb GCACCACAGCTGTCAGCTGTTTCAATAAGCAATTTACT------CGGCCAAAGCTTTGCA 684

Zm GCACCACAGTTGTCAGCTGTTTCAATAAGCAATTTGCT------CGGCCAAAGCTTTGCA 981

Hv GCACCACAGCTTTCAGCTGTTTCAATAAGCAGTTTGCT------TGGCCAGAGCTTTGCA 675

Tt GCACCACAGCTTTCAGCTGTTTCAATAAGCAGTTTGCT------TGGTCAGAGCTTTGCA 2286

Ta GCACCACAGCTTTCAGCTGTTTCAATAAGCAGTTTGCT------TGGTCAGAGCTTTGCA 690

Tm GCACCACAGCTTTCAGCTGTTTCAATAAGCAGTTTGCT------TGGTCAGAGCTTTGCA 826

Os1 GCACCACATCTTTCAGCCGTTTCTATAAGCAATCTCCT------TGGCCAGAGCTTTGCA 973

Os2 GCACCACATCTTTCAGCCGTTTCTATAAGCAATCTCCT------TGGCCAGAGCTTTGCA 1064

Al GCTTTTGACCTAGCAGATATGACTGCCTACAAAAATCTGGGTAATGGTGCAAGTTCT--- 874

Lt GCTTTTGACCTAGCAGATATGACTGCCTACAAAAATCTGGGTAATGGTGCAAGTTCT--- 874

AtMYB33 ACTTTTGATCTAGCAGATATGACTGCCTACAAAAATATGGGTAACTGTGCAAGTTCT--- 645

AtMYB65 GCTTCTGACCTATCAGATTTGGTTGCATGCAACATGCTAGGAACTGGCGCAAGTTCT--- 681

Rc GTCCCTGAACTTGCTGATATTACTACAAGCAGCATGCTGATAAAGGGTCTGAGTTCT--- 1052

Pt GTCCCCGAACTTCCTGATATTAGTGCAAGTAGCATTCTGATGAAAGGTCTGTGCTCT--- 669

* * * * * * * * * *

Sb TCAAAAAACTGTAGCTTCATGGATCAGGTAGACCA--AGCAGGGATG-CTGAAACAATCT 741

Zm TCAAAAAGTTGTAGCTTCATGGATCAGGTTGACCA--AGCGGGGATG-CTGAAACAATCT 1038

Hv TCCAAAAACTGCGGCTTCATGGATCAAGTAAACCA--AGCAGGGATG-CTAAAACAGTCT 732

Tt TCCAAAAACTGCGGCTTCATGGATCAAGTAAACCA--AGCAGGGATG-CTAAAACAGTCT 2343

Ta TCCAAAAACTGCGGCTTCATGGATCAAGTAAACCA--AGCAGGGATG-CTAAAACAGTCT 747

Tm TCCAAAAACTGCGGCTTCATGGATCAAGTAAACCA--AGCAGGGATG-CTAAAACAGTCT 883

Os1 TCAAAAAGCTGTAGCTTCATGGATCAGGTAAACCA--GACAGGGATG-CTAAAACAGTCT 1030

Os2 TCAAAAAGCTGTAGCTTCATGGATCAGGTAAACCA--GACAGGGATG-CTAAAACAGTCT 1121

Al CCTCGATATGAAAACTTCATGACACCAATAATGCCCTCCTCGAAGCGACTTTGGGAATCT 934

Lt CCTCGATATGAAAACTTCATGACACCAATAATGCCCTCCTCGAAGCGACTTTGGGAATCT 934

AtMYB33 CCTCGATATGAAAACTTCATGACACCAACAATCCCCTCCTCGAAGCGACTTTGGGAATCT 705

AtMYB65 TCCCGGTATGAGAGCTACATGCCACCAATATTGCCTTCCCCAAAGCAAATCTGGGAATCT 741

Rc TCTCAATATGGTAGCTTCATGTCACCATCAGCACATCGTCAGAAGCGTCTTCGAGAATCA 1112

Pt TCTCAATATGGAAGCTTCATGTCACCAACAATGCACCGTCAGAAGCGTCTTCGAGAGGCA 729

* * **** * * * * *

Sb AGCTGTGTGCTTCCTGCATTGAGCGATGCCATTGACGGTGTGCTTTCTTCAGTTGATCAA 801

Zm GGCTGTGTGCTTCCTGCATTGAGCGATGCCATTGACAGTGTGCTTTCCTCAGCTGATCAT 1098

Hv GACCCTTTACTCCCTGGATTGAGCGACACCATCAATGGCGCGCTCTCCTCGGTCGATCAA 792

Tt GACCATTTACTCCCTGGATTGAGTGACACCATCAATGGCGCGCTCTCCTCGGTCGATCAG 2403

Ta GACCCTTTACTCCCTGGATTGAGCGACACCATCAATGGCGCGCTCTCCTCGGTCGATCAG 807

Tm GACCCTTTACTCCCTGGATTGAGCGACACCATCAATGGCACGCTCTCCTCGGTCGATCAG 943

Os1 GATGGTGTGCTTCCTGGATTGAGCGATACCATCAACGGTGTGATTTCCTCGGTGGATCAA 1090

Os2 GATGGTGTGCTTCCTGGATTGAGCGATACCATCAACGGTGTGATTTCCTCGGTGGATCAA 1181

Al GAATTGTTGTATCCTGGGTGTAGCAGTACCGTAAAGCAAGAATTCTTGTCGCCTGAACAA 994

Lt GAATTGTTGTATCCTGGGTGTAGCAGTACCGTAAAGCAAGAATTCTTGTCGCCTGAACAA 994

AtMYB33 GAGTTGTTGTATCCTGGGTGTAGCAGTACCATAAAGCAAGAATTCTCGTCGCCTGAACAA 765

AtMYB65 GGATCTCGGTTTCCCATGTGCAGCAGTAACATAAAGCATGAATTCCAATCGCCGGAACAC 801

Rc ACAACTTTTATACCTGGTTACAGTGGCAATATAAAAAGCGAATTCCCTTTGTTTGACCAA 1172

Pt ACAACCTTATTATCCAGTTTCAGTGGTGGCATGAAAAATGATTTCCACTTGTTTGACCAG 789

* * * * ** **

Sb TTTTCAAATGACTCT---GAGAAGCTCAAGCAAGCTTTAGGTTTTGATTATCTTAATGAA 858

Zm TTTTCAAATGACTCT---GAGAAGCTCAGGCAGGCTTTAGGTTTTGATTATCTGAATGAA 1155

Hv TTCTCAAATGACTCT---GAGAAGCTCAAGCAGGCTCTTGGTTTTGACTATCTCCACGAA 849

Tt TTCCCAAATGACTCT---GAGAAGCTCAAGAAGGCTCTGGGTTTTGACTATCTCCACGAA 2460

Ta TTCTCAAATGACTCT---GAGAATCTCAAGAAGGCTCTGGGTTTTGACTATCTCCATGAA 864

Tm TTCTCAAATGACTCT---GAGAATCTCAAGAAGGCTCTGGGTTTTGACTATCTCCATGAA 1000

Os1 TTCTCAAATGACTCT---GAGAAGCTCAAGCAGGCTGTGGGTTTTGACTATCTCCATGAA 1147

Os2 TTCTCAAATGACTCT---GAGAAGCTCAAGCAGGCTGTGGGTTTTGACTATCTCCATGAA 1238

Al TTCCAGAACACATCTCCACAAAAGATTTCCAAAACTTGCAGTTTCTCAGTTCCTTGTGAT 1054

Lt TTCCAGAACACATCTCCACAAAAGATTTCCAAAACTTGCAGTTTCTCAGTTCCTTGTGAT 1054

AtMYB33 TTCCGGAACACATCTCCACAAACGATTTCCAAAACTTGCAGCTTCTCAGTTCCTTGTGAT 825

AtMYB65 TTTCAGAATACGGCTGTACAGAAGAATCCCAGATCTTGCAGTATCTCG---CCTTGTGAT 858

Rc TTTCAGGATGAATCTTGTGATAAAGTTGCCCAATCTTTTGGGTTATCTTTTCCATTTGAT 1232

Pt TTTCAGGATG------GTGATAAAGCTGCTCAATACTTTGGGTTATCTTTTCCATTTGAT 843

* * * * * * * **

Sb GCCAA---TGCTAGCAGCAAGAGTATTGCACCTTTCGG---GGTTGCACTTACTGGCAGC 912

Zm GCCAA---TGCTAGCAGCAAGAGTATTGCACCTTTCGG---GGTTGCACTTACTGGCAGC 1209

Hv GCCAA---CTCTAGCAGCAAGATTATTGCACCATTTGG---GGGTGCGCTTACTGGCAGC 903

Tt GCCAA---CTCTAGCAGCAAGACTATTGCACCATTTGG---GGGTGCACTTACTGGCAGC 2514

Ta GCCAA---CTCTAGCAGCAAGATGATTGCACCATTTGG---GGGTACACTTACTGGCAGC 918

Tm GCCAA---CTCTAGCAGCAAGATGATTGCACCATTTGG---GGGTGCACTTACTGGCAGC 1054

Os1 GCCAA---CTCTACCAGCAAGATTATTGCACCTTTCGG---GGGTGCACTTAATGGCAGC 1201

Os2 GCCAA---CTCTACCAGCAAGATTATTGCACCTTTCGG---GGGTGCACTTAATGGCAGC 1292

Al GTTGAGCATCCTCTCTATGGAAACCGACATTCACCT------ATTATGATTTCAGATAGC 1108

Lt GTTGAGCATCCTCTCTATGGAAACCGACATTCACCT------ATTATGATTTCAGATAGC 1108

AtMYB33 GTTGAGCATCCTCTCTATGGAAACCGACATTCACCT------GTTATGATTCCAGATAGC 879

AtMYB65 GTTGATCATCATCCTTATGAAAACCAACATTCGTCTCATATGATGATGGTTCCTGATAGC 918

Rc CCTGA---TCC---CACCAAGAACCCTCAGTCCTTT------GGTGAAAATCAGGGCAGC 1280

Pt CCTGA---TTCTGCCACCAAGAACCCAGAGATTTTT------GGTGAAAATCAGGGTAGC 894

* * ***

Sb CATGCCTTTCTAAATGGCAACTTCTCTGCTTCTAGGCCCACAAATGGTCCTTTGAAGATG 972

Zm CATGCCTTTTTAAATGGCAATTTCTCTGCTTCTAGGCCCACAAATGGTCCTTTGAAGATG 1269

Hv CATGCCTTTTTAAATGGCACCTTCTCTACTTCTAGGACCATCAATGGTCCTTTGAAGATG 963

Tt CATGCCTTTTTAAATGGCACCTTTTTTACTTCTAGGACCATCAATGGTCCTTTGAAGATG 2574

Ta CATGCCTTTTTAAATGGCACCTTCTCTACTTCTAGGACCATCAATGGCCCTTTGAAGATG 978

Tm CATGCCTTTTTAAATGGCACCTTCTCTACTTCTAGGACCATCAATGGTCCTTTGAAGATG 1114

Os1 CATGCCTTTTTAAATGGCAATTTCTCTGCTTCTAGGCCCACAAGTGGTCCTTTGAAGATG 1261

Os2 CATGCCTTTTTAAATGGCAATTTCTCTGCTTCTAGGCCCACAAGTGGTCCTTTGAAGATG 1352

Al CATACCCCTACGGATGGCATTGTTCCTTCTTCTAAGCCCTCATATGGGGCAGTGAAGCTG 1168

Lt CATACCCCTACGGATGGCATTGTTCCTTCTTCTAAGCCCTCATATGGGGCAGTGAAGCTG 1168

AtMYB33 CATACCCCTACGGATGGCATTGTTCCTTATTCTAAGCCCTTATATGGGGCAGTGAAGCTG 939

AtMYB65 CATACAGTTACGTATGGCATGCATCCTACTTCTAAGCCCTTGTTTGGGGCAGTGAAGCTG 978

Rc CATACCCTTGCAAATGGCGATTTCTCTACTTCCAAGCTCACCTCTGGGCCTGTGAAGTTG 1340

Pt CATACCCTTGCTAATGGCGATTTCTCTGCTTCTAAGCCCACTTCAGAGGCTGTGAAGTTT 954

*** * * ***** * *** * * * * * ***** *

Sb GAGCTCCCTTCACTCCAAGATACTGAATCTG----ATCCAAATAGCTGGCTCAAGTATAC 1028

Zm GAGCTCCCTTCACTCCAAGATACTGAATCTG----ATCCAAATAGCTGGCTCAAGTATAC 1325

Hv GAGCTCCCTTCACTCCAAGATACCGAATCTG----ATCCGAATAGCTGGCTCAAGTATAC 1019

Tt GAGCTCCCTTCACTCCAAGATACCGAATTTG----ATCCGAATAGCTGGCTCAAGTATAC 2630

Ta GAGCTCCCTTCACTCCAAGATACCGAATCTG----ATCCGAATAGCTGGCTCAAGTATAC 1034

Tm GAGCTCCCTTCACTCCAAGATACCGAATCTG----ATCCGAATAGCTGGCTCAAGTATAC 1170

Os1 GAGCTCCCTTCACTCCAAGATACTGAATCTG----ATCCAAACAGCTGGCTCAAGTACAC 1317

Os2 GAGCTCCCTTCACTCCAAGATACTGAATCTG----ATCCAAACAGCTGGCTCAAGTACAC 1408

Al GAGCTCCCTTCATTCCAATATTCAGAAACAA---CATTTGACCAG-TGG----AAGAAAT 1220

Lt GAGCTCCCTTCATTCCAATATTCAGAAACAA---CATTTGACCAG-TGG----AAGAAAT 1220

AtMYB33 GAGCTCCCTTCATTCCAATATTCAGAAACAA---CATTTGACCAG-TGG----AAGAAAT 991

AtMYB65 GAGCTCCCTTCATTCCAATATTCAGAAACTAGTGCATTTGATCAG-TGG----AAGACGA 1033

Rc GAGCTCCCTTCACTCCAATATCCAGAAACTG----ATTTAGGTAGCTGG----GGTACAT 1392

Pt GAGCTCCCTTCACTCCAATATGCAGAAACTG----ATTTAGGTGGCTGG----GGGGCAT 1006

************ ***** ** * *** ** * ***

Sb TGTGGCTCCTGCAATGC---AGCCTACT-GAGTTAGTAGATCCTTACCTGCAGTCTCCAT 1084

Zm TGTGGCTCCTGCAATGC---AGCCTACT-GAATTAGTAGATCCTTACCTGCAGTCTCCAT 1381

Hv CGTGGCTCCTGCGATGC---AGCCTACG-GAGTTGGTTGATCCGTACCTGCAGTCCCCGA 1075

Tt CGTGGCTCCTGCGATGC---AGCCTACG-GAGTTGGTTGATCCCTACCTGCAGTCTCCGA 2686

Ta CGTGGCTCCTGCGATGC---AGCCTACG-GAGTTGGTTGATCCCTACCTTCAGTCTCCGA 1090

Tm CGTGGCTCCTGCGATGC---AGCCTACG-GAGTTGGTTGATCCCTACCTTCAGTCTCCGA 1226

Os1 TGTAGCTCCTGCGTTGC---AGCCTACT-GAGTTAGTTGATCCCTACCTGCAGTCTCCAG 1373

Os2 TGTAGCTCCTGCGTTGC---AGCCTACT-GAGTTAGTTGATCCCTACCTGCAGTCTCCAG 1464

Al CGTCATCTCCTCCACACTCTCATCTTCTTGATCCCTTTGATACTTACATTCAGTCTCCAC 1280

Lt CGTCATCTCCTCCACACTCTCATCTTCTTGATCCCTTTGATACTTACATTCAGTCTCCAC 1280

AtMYB33 CGTCATCTCCTCCACACTCTGATCTCCTTGATCCCTTTGATACTTACATTCAGTCTCCAC 1051

AtMYB65 CTCCGTCACCTCCACACTCAGATCTCCTTGACTCTGTTGATGCCTATATTCAATCTCCAC 1093

Rc C------CCCACCACC------TTTACTTGAGACTCTTGACAATTTCATCCAGTCTCCAC 1440

Pt CTTGTTCCCCATCACC------TTTAATCGAGTCTGTTGATACTTTTATTCAATCTCCTC 1060

* * ** * ** * * ** ** **

Sb CAGCG---ACCCCTTCAGTGAAATCTGAGT---GTGCATCGCCGAGGAACAGTGGTCTTT 1138

Zm CAGCG---ACCCCTTCAGTGAAATCTGAGT---GTGCATCGCCGAGGAACAGTGGTCTTT 1435

Hv CAGCA---ACTCCGTCAGTGAAATCGGAGT---GTGCTTCGCCGAGGAACAGCGGCCTCT 1129

Tt CAGCA---ACTCCGTCAGTGAAATCGGAGT---GTGCGTCGCCAAGGAACAGCGGCCTCT 2740

Ta CAGCA---ACTCCGTCAGTGAAGTCGGAGT---GTGTGTCGCCAAGGAACAGCGGTCTCT 1144

Tm CAGCA---ACTCCGTCAGTGAAGTCGGAGT---GTGTGTCGCCAAGGAACAGCGGTCTCT 1280

Os1 CAGCA---ACCCCTTCAGTGAAATCAGAGT---GCGCGTCGCCAAGGAATAGTGGCCTTT 1427

Os2 CAGCA---ACCCCTTCAGTGAAATCAGAGT---GCGCGTCGCCAAGGAATAGTGGCCTTT 1518

Al CACCACCTACGGGGAGAGAAGAGTCAGATT---TATATTCAAGTTTTGATACTGGTCTGC 1337

Lt CACCACCTACGGGGAGAGAAGAGTCAGATT---TATATTCAAGTTTTGATACTGGTCTGC 1337

AtMYB33 CACCACCAACGGGGGGAGAAGAGTCAGATT---TATATTCAAATTTTGATACTGGTCTGC 1108

AtMYB65 CACCATC-GCAGGTAGAG--GAGTCAGATTGTTTCTCTTCA---TGCGACACCGGCCTAC 1147

Rc CA------ACAGCTATAATTGAATC---TT---CTCCA------CGTAATAGTGGCCTCT 1482

Pt CT------ACTGGGACAGTCGAGTCAAATT---TTCCATCACCACGTAATAGTGGGCTAT 1111

* * * * * * * * ** **

Sb TGGAAGAGCTGCTTCATGAAGCTCAGGCACTAAGATCTG---GGAAGAACCAACAACCAT 1195

Zm TGGAAGAGCTGCTTCATGAAGCTCAGGCACTAAGATCTG---GGAAGAACCAACAATCAT 1492

Hv TGGAAGAGCTGCTTCATGAAGCTCAGGGACTAAGATCTG---GGAAGAATCAGCAGCTCT 1186

Tt TGGAAGAGCTGCTTCATGAAGCTCAGGGACTAAGATCTG---GGAGGAACCAGCAGCTTT 2797

Ta TGGAAGAGCTGCTTCATGAAGCTCAGGGACTAAAATCTG---GGAAGAATCAGCAGCTTT 1201

Tm TGGAAGAGCTGCTTCATGAAGCTCAGGGACTAAAATCTG---GGAAGAATCAGCAGCTTT 1337

Os1 TGGAAGAGTTGATTCATGAAGCTCAGACCCTAAGATCCG---GGAAGAACCAACAGACAT 1484

Os2 TGGAAGAGTTGATTCATGAAGCTCAGACCCTAAGATCCG---GGAAGAACCAACAGACAT 1575

Al TCGATATGTTGCTTCTGGAGGCCAAGATCAGAAATAATACTACAAAGAACAATT---TGT 1394

Lt TCGATATGTTGCTTCTGGAGGCCAAGATCAGAAATAATACTACAAAGAACAATT---TGT 1394

AtMYB33 TCGATATGTTGCTTCTGGAAGCCAAGATCAGAAATAATAGTACAAAGAACAATT---TGT 1165

AtMYB65 TAGATATGTTACTTCATGAGGCCAAGATCAAAACTAGTG---CGAAGCACAGTT---TGT 1201

Rc TGGATGCTTTACTTTATGAGGCTAAAGCTTTAAGCAGTG---CTAAGAATCATT---CAT 1536

Pt TGGATGCTTTACTTTACGAGGCCAGAACTCTAAGCAGTG---CAAAGAATCAAT---CAT 1165

* ** * * ** ** ** * * * *

Sb CGGT------------CCGAAGTTCAAGTTCTTCTGCTGGCACACCTTGTGAGACTAC-T 1242

Zm CGGT------------CCGAAGTTCAAGTTCTTCTGCTGGCACACCTTATGAGACTAC-C 1539

Hv CCGT------------GAGAAGTTCAAGTTCCTCTGTCAGCACGCCGTGTGA---TAC-C 1230

Tt CCGT------------GAGGAGTTCAAGTTCCTCTGTCAGCACGCCGTGTGA---TAC-C 2841

Ta CCGT------------GAGAAGTTCAAGTTCCTCTGTCAGTACGCCGTGTGA---TAC-T 1245

Tm CCGT------------GAGAAGTTCAAGTTCCTCTGTCAGTACGCCGTGTGA---TAC-T 1381

Os1 CTGT------------GATAAGTTCTAGTTCTTCTGTCGGTACGCCATGTAA---TAC-T 1528

Os2 CTGT------------GATAAGTTCTAGTTCTTCTGTCGGTACGCCATGTAA---TAC-T 1619

Al ---------------ACAAGAGCTGCGCTTCA---ACTATTCCATCAGCTGATCTTGG-C 1435

Lt ---------------ACAAGAGCTGCGCTTCA---ACTATTCCATCAGCTGATCTTGG-C 1435

AtMYB33 ---------------ACAGGAGCTGCGCTTCA---ACTATTCCATCAGCTGATCTTGG-C 1206

AtMYB65 TGATGTCATCACCCCAGAAGAGTTTCAGTTCA---ACTACTTGCACGACCAATGTTACTC 1258

Rc CGG------------AGAAGAGTTCAAATTCATCAACTGTTACTCCTGGTGAACTTGCTG 1584

Pt CTG------------ATAAGAGTTCAAATTCATCTACCATTACTCCTGGTGACAATGCAG 1213

** *** * *

Sb ACGGTGGTTAGCCCAGAGTTTGATATGGGCCAG----------GAATATTGGGAAGAACA 1292

Zm ACGGTGGTTAGCCCAGAGTTTGATATGGGTCAG----------GAATATTGGGAAGAACA 1589

Hv ACGGTGGTTAGCCCGGAGTTTGATCTCTGTCAG----------GAATATTGGGAAGAACG 1280

Tt ACGGTGATTAGCCCAGAG---GATCTCTGTCAG----------GAATATTGGGAAGAACG 2888

Ta ACGGTGGTTAGCCCAGAGTTTGATATCTGTCAG----------GACTATTGGGAAGAACC 1295

Tm ACGGTGGTTAGCCCAGAGTTTGATATCTGTCAG----------GACTACTGGGAAGAACC 1431

Os1 ACGGTTCTTAGCCCAGAGTTTGATATGTGTCAG----------GAATACTGGGAAGAACA 1578

Os2 ACGGTTCTTAGCCCAGAGTTTGATATGTGTCAG----------GAATACTGGGAAGAACA 1669

Al AAGGT---------------TACTGTGTCCCAA----------ACTAAATCCGAGGATTT 1470

Lt AAGGT---------------TACTGTGTCCCAA----------ACTAAATCCGAGGATTT 1470

AtMYB33 CAGGT---------------TACTGTATCCCAA----------ACTAAATCCGAGGAGTT 1241

AtMYB65 AGAATG--------------TACCACGTGGCAGCGAAAACCTGATCAAATCAGGAGAATA 1304

Rc AATGTTCT--GCCA-----TTAATATTTGTGAG----------ACAGAATGGGAAGATTA 1627

Pt ACTGTTCT--GCCC-----TTAATATTAGCGAG----------ACAGAATGGGAAGACTA 1256

* * * * * **

Sb GC---CTGGTTCTTTCCTCAGTGAATATGCT---CATTTTAGTGGAAATTCTTTGACC-- 1344

Zm GC---CCAGTTCTTTCCTCAGTGAATATGCT---CATTTTAGTGGAAATTCTTTCACT-- 1641

Hv ---------------TCTGAATGAATATGCC---CCATTCAGTGGCAATTCACTCACT-- 1320

Tt ---------------TCTGAATGAGTATGCTGCTCCCTTCAGTGGTAATTCACTCACT-- 2931

Ta ---------------TCTGAATGAATATGCT---CCTTTCAGTGGCAATTCACTCACT-- 1335

Tm ---------------TCTGAATGAATATGCT---CCTTTCAGTGGCAATTCACTCACT-- 1471

Os1 ACATCCTGGTCCATTCCTCAATGACTGTGCT---CCTTTCAGTGGCAATTCATTCACT-- 1633

Os2 ACATCCTGGTCCATTCCTCAATGACTGTGCT---CCTTTCAGTGGCAATTCATTCACT-- 1724

Al ------------------TGACAATTCCCTTAAGAGTTT---GGTCCATTCCGACAT--- 1506

Lt ------------------TGACAATTCCCTTAAGAGTTT---GGTCCATTCCGACAT--- 1506

AtMYB33 ------------------TGACAATTCCCTTAAGAGCTTCTTGGTTCATTCCGAAAT--- 1280

AtMYB65 ------------------TGAAGATTCCCAAAAGTATTT---GGGTCGCTCCGAGATT-- 1341

Rc ------------------TGGTGATCCCCTTTCCCCATT---GGGTCACACTGCGACTTC 1666

Pt ------------------TGGTGATCCCATTTCTCCTTT---GGGTCATCCTGCAGCTTC 1295

* ** * *

Sb ----------GAATCCACTCCT-------GTTAGTGCTGCGTCACCTGATTTCTTTCAGT 1387

Zm ----------GAATCCACTCCTC----CTGTTAGTGCTGCGTCACCTGATATCTTCCAGC 1687

Hv ----------GGATCCACCGCTC----CTATGAGTGCTGCGTCGCCTGATGTTTTTCAGC 1366

Tt ----------GGATCCACTGCTC----CTGTGAGCGCTGCGTCGCCTGATGTTTTTCAGC 2977

Ta ----------GGATCTACGGCTC----CTGTTAGCGCTGCGCCGCCTGATGTTTTTCAGC 1381

Tm ----------GGATCCACGGCTC----CTGTTAGCGCTGCGTCGCCTGATGTTTTTCAGC 1517

Os1 ----------GAATCCACCCCTC----CTGTTAGCGCTGCATCGCCTGACATCTTTCAGC 1679

Os2 ----------GAATCCACCCCTC----CTGTTAGCGCTGCATCGCCTGACATCTTTCAGC 1770

Al ------------GTCCA-CACAA---AATGCAGATGGAATTCCACCA------------- 1537

Lt ------------GTCCA-CACAA---AATGCAGATGGAATTCCACCA------------- 1537

AtMYB33 ------------GTCCA-CACAA---AATGCAGATGAAACTCCACCA------------- 1311

AtMYB65 --------ACAAGTCCCTCGCAACTTAGTGCAGGTGGTTTTTCATCAG------------ 1381

Rc TCTTTTCAGTGAGTGCACTCCTA-TAAGTGCCAGTGGAAGTTCTTTGGATGAACCACCAC 1725

Pt CCTTTTCAGCGAGTGCACTCCCA-TCAGTGCCAGTGGAAGCTCTTTGGATGAATCACCAC 1354

* * * *
